# Supplementary material for: Perceived discrimination and contextual problems among children and adolescents in northern Chile
Source: PLoS One. 2021 Feb 19;16(2):e0246998. doi: 10.1371/journal.pone.0246998 (PMC7894936; doi:10.1371/journal.pone.0246998)
Supplement: S2 File — (DOCX) [file pone.0246998.s002.docx]

**Imputation Primary**

TITLE: **IMPUTATION PLOSONE PRIMARIA**

DATA: FILE IS MATCH PRIMARIA TOT PLOSONE5 NOV.dat;

VARIABLE: NAMES=

CodCole AGE GENDER PD_FAM PD_ESC PD_COM

ID_ETNIC ACOLECT EDST VULNERAB;

USEVARIABLES=

CodCole AGE GENDER PD_FAM PD_ESC PD_COM

ID_ETNIC ACOLECT EDST VULNERAB;

MISSING= ALL (999);

DATA IMPUTATION:

IMPUTE= EDST;

NDATASET= 7;

SAVE= MISSIPLOPR5NOV*.DAT;

ANALYSIS: TYPE= BASIC;

OUTPUT: TECH8;

**Syntax primary PATH**

DATA: FILE IS MISSIPLOPR5NOVlist.DAT;

TYPE = IMPUTATION ;

VARIABLE: NAMES ARE

CodCole AGE GENDER PD_FAM PD_ESC PD_COM

ID_ETNIC ACOLECT EDST VULNERAB;

MISSING = ALL(999);

WITHIN = AGE GENDER EDST ID_ETNIC ACOLECT

PD_ESC;

BETWEEN = VULNERAB ;

CLUSTER = CodCole ;

ANALYSIS: Type = twolevel ;

H1iterations = 10000 ;

mconv=0.000001 ;

MODEL:

%within%

PD_FAM ON

ACOLECT EDST ;

PD_ESC ON

ACOLECT EDST GENDER AGE ;

PD_COM ON

EDST ;

ACOLECT ON EDST ;

ACOLECT ON ID_ETNIC ;

%Between%

PD_FAM ON VULNERAB ;

PD_COM ON VULNERAB ;

MODEL INDIRECT:

PD_FAM IND ID_ETNIC ;

PD_FAM IND EDST ;

PD_ESC IND ID_ETNIC ;

PD_ESC IND EDST ;

ANALYSIS: ESTIMATOR = MLR;

OUTPUT: STDYX TECH1 TECH4 TECH8 TECH9 sampstat;

**Imputation secondary**

TITLE: IMPUTATION PLOSONE SECUNDARIA

DATA: FILE IS MATCH Secundaria TOT PLOSONE4 EXC.dat;

VARIABLE: NAMES=

CodCole AGE GENDER PD_FAM PD_ESC PD_COM

AUCOLECT ID_ETNIC EDS VULNERAB;

USEVARIABLES=

CodCole AGE GENDER PD_FAM PD_ESC PD_COM

AUCOLECT ID_ETNIC EDS VULNERAB;

MISSING= ALL (999);

DATA IMPUTATION:

IMPUTE= EDS;

NDATASET= 7;

SAVE= MISSIPLOS4EX*.DAT;

ANALYSIS: TYPE= BASIC;

OUTPUT: TECH8;

**Syntax secondary PATH**

DATA: FILE IS MISSIPLOS4EXlist.DAT;

TYPE = IMPUTATION ;

VARIABLE: NAMES ARE

CodCole AGE GENDER PD_FAM PD_ESC PD_COM

AUCOLECT ID_ETNIC EDS VULNERAB;

MISSING = ALL(999);

WITHIN = AGE GENDER EDS ID_ETNIC AUCOLECT

PD_COM PD_ESC;

BETWEEN = VULNERAB ;

CLUSTER = CodCole ;

ANALYSIS: Type = twolevel ;

H1iterations = 10000 ;

MODEL:

%within%

PD_FAM ON

AUCOLECT ID_ETNIC EDS GENDER ;

PD_ESC ON

ID_ETNIC EDS GENDER ;

PD_COM ON

EDS GENDER ;

AUCOLECT ON EDS AGE ;

AUCOLECT ON ID_ETNIC ;

%Between%

PD_FAM ON VULNERAB ;

MODEL INDIRECT:

PD_FAM IND ID_ETNIC ;

PD_FAM IND EDS ;

PD_FAM IND AGE ;

ANALYSIS: ESTIMATOR = MLR;

OUTPUT: STDYX TECH1 TECH4 TECH8 TECH9 sampstat;
