# Supplementary material for: Perceived discrimination and contextual problems among children and adolescents in northern Chile
Source: PLoS One. 2021 Feb 19;16(2):e0246998. doi: 10.1371/journal.pone.0246998 (PMC7894936; doi:10.1371/journal.pone.0246998)
Supplement: Attachment — Submitted filename: Recommendations PLOS.pdf [file pone.0246998.s005.pdf]

Dear authors.

It's a well written paper, which talk about a complex problem which do exist around the world. I consider that you have not big problems with your article.

First of all, I recommend just a little explanation about used instruments: you must explain (one by one) if it have a Chilean validation and describe metric properties too.

Second, clarify about one finding: you said (line 262 to 263) that ethnic identity does not correlate with peers problems and in the chart below the findings are contradictory.

Third, and last, I would like you to talk a little more about a possible intervention.
