# Supplementary material for: Perceived discrimination and contextual problems among children and adolescents in northern Chile
Source: PLoS One. 2021 Feb 19;16(2):e0246998. doi: 10.1371/journal.pone.0246998 (PMC7894936; doi:10.1371/journal.pone.0246998)
Supplement: Attachment — Submitted filename: PONE-D-20-05463_R1.pdf [file pone.0246998.s007.pdf]

PLOS ONE

Perceived discrimination and contextual problems among children and adolescents in Northern Chile

--Manuscript Draft--

**Manuscript Number:**	PONE-D-20-05463R1
**Article Type:**	Research Article
**Full Title:**	Perceived discrimination and contextual problems among children and adolescents in Northern Chile
**Short Title:**	Perceived discrimination and contextual problems among children and adolescents in Northern Chile
**Corresponding Author:**	Jerome Aníbal Flores, Ph.D<br>Centro de Justicia Educacional. Universidad de Tarapaca<br>Arica, Arica y Parinacota Chile CHILE
**Keywords:**	perceived discrimination; ethnic identity; collective self-esteem; children and adolescents
**Abstract:**	Discriminatory behaviors among inter-ethnical relations in schools have long been noted and studied, but there are several correlations between discriminatory behaviors and other constructs that need further investigation. As an example, the relation between perceived discrimination and contextual problems—which include family, school and peer problems—among children and adolescents in Latin America has received little attention from previous studies. Further, the mediating role of ethnic identity and collective self-esteem in this relation also needs to be considered as they could be proven as protective factors for discriminatory behavior and its outcomes. Therefore, this study aimed to, first, establish the effects of the relation between perceived discrimination and contextual problems in inter-ethnic students aged 8-19 years living in Arica, Chile; and second, to identify the role that ethnic identity and collective self-esteem play within this relation. In order to investigate this matter, a cross-sectional study was carried out with 3700 students in 29 schools between the fourth year of primary education and the last year of secondary education, aged between 9-18 years, with 48.4% men and 51.6% women. The sample was divided into primary and secondary groups. The scales utilized were the Everyday Discrimination Scale, Multi-Group Ethnic Identity Measure-Revised Scale, Collective Self-Esteem Scale and the dimensions of contextual family, school and peer problems , as well as the general index of contextual problems of the Child and Adolescent Assessment System. For data analysis, four models of structural equations were proposed to account for the relations between variables, two in each group, and all were optimally adjusted. We found that perceived discrimination and ethnic identity had a direct effect on contextual problems in general (.38-.45) and when separated (.29-.39), and collective self-esteem had only a mediating role. This study showed that strategized interventions focusing on ethnic identity and perceived discrimination should be utilized by schools to create a better developing environment.
**Order of Authors:**	Jerome Aníbal Flores, Ph.D
	Alejandra Caqueo-Urízar
	Alfonso Urzua
	Lirna Quintana
	Matías Irarrázaval
**Opposed Reviewers:**	
**Response to Reviewers:**	Dear Editor and reviewers:<br><br>We are grateful of all comments. We decide to divide the sample in two groups, primary and secondary, since we used different SENA age version in each one. Even if both SENA version share more than 70% items, slight differences in the rest can affect the analysis, and the secondary version has one item more.

Powered by Editorial Manager® and ProduXion Manager® from Aries Systems Corporation

1.- Please, add empirical information in the abstract.

It was adding now

2.- Please, add an operative definition of the main constructs used.

There is not a cut-off point for to establish enough collective self-esteem or ethnic identity. Then it was not specified. SENA has cut-off for four risk zones, but it was not used because a continuous variable can show clearer the relationship.

3.- In English, a decimals point is used, not comma.

It was changed

4.- Please, add the main hypothesis at the end of the introduction.

We added three hypotheses

5.- Add more information about sample and sampling procedure as well as others variables like: % by age, socio-economic level, IQ, previous mental disorders, etc. Add all the sample information in the method
section, not as a results.

It was added all information and change the position in the document. We don't ask socioeconomic level to child's because the primary group probably don't know

6.- Do you have any information about non-response? Describe inclusion/exclusion criteria if part of the data was excluded from the analysis.

We added criteria

7.- Were outliers removed from the data? Which method did you use to deal with missing data in the analyses? What variables are related to missing data?

We identified out-layers in both groups, but we dint remove. We explain the differences between include or not at the end of result section.
The effects of removing the out-layers are considered. The vast majority of the relationships are maintained and vary in a range of 3-5%. Only one relationship ceases to be significant, ethnic identity on school problems in primary group. In sum, this means that relationships are not generated by the presence of out-layers. In any case, by removing them and recalculating the Z-scores, new out-layers appear. Since the MLR estimator that is resistant to non-normality has been chosen, it is decided to keep all the cases in the sample.

8.- Add information about the psychometric properties of the measures used in this study. In addition, add Omega values, not Cronbach. Cronbach alpha has a lot of limitations from a psychometric point of view.

We added all information about each scale and omega indices for all

9.- Add subheadings in the results section (as function of the goals).
We added subheadings in this section

10.- Add more information about mediation analyses performed.

We added all information

11.- Please, before to SEM analysis, compute a MANOVA or MANCOVA in mean comparisons by gender and age. Add effect sizes in mean comparisons.

We added a MANOVA and effect sizes

12.- Please, add other godness of fit indices, eg.: TLI, BIC, IC RMSEA 90%, etc. Add

information about
goodness of fit indices. Test model assumptions (e.g., normality).

We added information about normality, and more fit indices

13.- Add more limitations (e.g., self-reports, no scale to test social desirability).

We added

14.- Check English grammar.

We reviewed it

Reviewer #1: The work entitled “Perceived discrimination and contextual problems among children and adolescents in Northern Chile" is of great interest. The research is very stimulating; it contains new scientific knowledge and provides comprehensive information for further development of this productive line of research. However, I have some comments to make that should be addressed before I
recommend this manuscript for publication.

- In the introduction, authors should consider devoting some lines about psychological problems in children and adolescence. With this regard recent relevant research analyzing prevalence of emotional and behavioural problems at those ages should be introduced (Dalsgaard et al., 2020; Ortuño-Sierra, Aritio-Solana, & Fonseca-Pedrero, 2018).

The inclusion of mental health studies diverts the focus from discrimination in relation to contextual problems. We have reviewed the articles, but they do not mention the contextual problems as to connect them with our article

- Authors should consider the following affirmation in the introduction: “Based on the aforementioned facts,
we believe this study could contribute significantly to reduce the effects of discrimination in schools within the northernmost regions of Chile and other applicable contexts”. First, this sentence would better fit in the discussion section. Second I am not completely sure about the adequacy of such affirmation considering the nature of the study. Authors may contribute relevant information to comprehend the nature of the problema and from that prevent discrimination but they dont know if that reduce significantly the effects of discrimination.

We rewording it to keep in the same section as part of relevance to investigate this topic

- In addition, the following sentence should be placed in the discussion section. “The aim of the study was achieved, as perceived discrimination and ethnic identity were regarded”. With this regard, the objectives section should provide information about hypothesis.

We removed that sentence.

- In the participants section, authors may consider including the age distribution.

We added it

- In the instruments section, authors please check
for grammatical mistakes in the following sentence: “It is a Likert-type scale consisted…”

We removed it

- In addition, authors should described the estimator when talking about evidences of internal consistency of the scores. Also, evidences of the MEIM-R are not provided.

Powered by Editorial Manager® and ProduXion Manager® from Aries Systems Corporation

We described MLR estimator in SEM, and added omega indices in each instrument

- Moreover, authors should consider explaining whether the instruments are self-reported or information could or have to be provided by parents, teacher and/or clinicals. This is relevant beacuse in the procedure it is not clear (please also consider modifying) if the students themselves completed the questionnaires or if the teachers or someone else provided information about the students.

We added it. Also, we expand procedure section

- With this regard, and assuming the students completed the questionnaires, have the authors consider that there are concerns raised about children under 11-10 years old answering self-reported questionnaires due to the lack of instrospection?

We explained that there are two SENA instruments. One for each group, specially address to be easy to understand and answer.

- Also, it seems after eading the procedure that the written consent was asked to the participants. I do not know how appropriate it is that under 18 and in this case children of 8,9,10 years old provide this kind of consent. In my opinion, parents, teachers or legal tutors should have asked for this aspect.

We added information in procedure section. First, we ask to parents. Then we ask student themselves. We think that is more respectful with the child's and adolescents.

- I would also consider making the objectives more specifics and related to the results and conclusions. It is not till the results and the discussion section when one start realizing why the authors includen the SEM analysis.

We added three hypotheses to connect objective with analysis and results

Please also explain in the data analysis section the reference values for the goodnes-of-fit índices values instead of doing it in the results section.

We moved it

- Finally, authors should consider revising the writing when talking about internal consistency or validity in some parts of the paper. First of all, validity is not a property of the test but inferences of the scores, and also, there are sources or validity evidences, as it is reflected in the APA standards. Attending to this approach it would be more appropriate to talk about evidences of internal structure or evidences of relation with other variables or external variables. In addition, the reliability is not a characteristic of the test. It is more correct to talk about reliability of the scores or estimation of the reliability of the scores (Prieto & Delgado, 2010).

We rewording it

Reviewer #2: Lines 176, 183, 200, 220: please indicate the metric properties of the instruments and clarify if they have validation in your country. Not only Spanish translation.

We added it

Lines 262 to 263: It is not clear why you say that there is no statistically significant correlation between ethnic identity and problems with peers. In the correlation chart the information is different.

It was non-significant correlation. We remarked this again.
r= ,020

Powered by Editorial Manager® and ProduXion Manager® from Aries Systems Corporation

	Thanks
**Additional Information:**	
**Question**	**Response**
**Financial Disclosure**<br><br>Enter a financial disclosure statement that describes the sources of funding for the work included in this submission. Review the submission guidelines for detailed requirements. View published research articles from *PLOS ONE* for specific examples.<br><br>This statement is required for submission and **will appear in the published article** if the submission is accepted. Please make sure it is accurate.<br><br>**Unfunded studies**<br>Enter: *The author(s) received no specific funding for this work.*<br><br>**Funded studies**<br>Enter a statement with the following details:<br>• Initials of the authors who received each award<br>• Grant numbers awarded to each author<br>• The full name of each funder<br>• URL of each funder website<br>• Did the sponsors or funders play any role in the study design, data collection and analysis, decision to publish, or preparation of the manuscript?<br>• **NO** - Include this sentence at the end of your statement: *The funders had no role in study design, data collection and analysis, decision to publish, or preparation of the manuscript.*<br>• **YES** - Specify the role(s) played.<br><br>* typeset	This research was funded by CONICYT-PIA-CIE160007
**Competing Interests**<br><br>Use the instructions below to enter a competing interest statement for this submission. On behalf of all authors, disclose any competing interests that could be perceived to bias this work—acknowledging all financial support and any other relevant financial or non-	The authors have declared that no competing interests exist

financial competing interests.<br><br>This statement **will appear in the published article** if the submission is accepted. Please make sure it is accurate. View published research articles from *PLOS ONE* for specific examples.<br><br>**NO authors have competing interests**<br><br>Enter: *The authors have declared that no competing interests exist.*<br><br>**Authors with competing interests**<br><br>Enter competing interest details beginning with this statement:<br><br>*I have read the journal's policy and the authors of this manuscript have the following competing interests: [insert competing interests here]*<br><br>* typeset	
**Ethics Statement**<br><br>Enter an ethics statement for this submission. This statement is required if the study involved:<br><br>• Human participants<br>• Human specimens or tissue<br>• Vertebrate animals or cephalopods<br>• Vertebrate embryos or tissues<br>• Field research<br><br>Write "N/A" if the submission does not require an ethics statement.<br><br>General guidance is provided below. Consult the submission guidelines for detailed instructions. **Make sure that all information entered here is included in the Methods section of the manuscript.**	The Ethics Committee of Universidad de Tarpacá approved this research. parental consent was in writing. student consent was in writing.

**Format for specific study types**<br><br>**Human Subject Research (involving human participants and/or tissue)**<br>• Give the name of the institutional review board or ethics committee that approved the study<br>• Include the approval number and/or a statement indicating approval of this research<br>• Indicate the form of consent obtained (written/oral) or the reason that consent was not obtained (e.g. the data were analyzed anonymously)<br><br>**Animal Research (involving vertebrate animals, embryos or tissues)**<br>• Provide the name of the Institutional Animal Care and Use Committee (IACUC) or other relevant ethics board that reviewed the study protocol, and indicate whether they approved this research or granted a formal waiver of ethical approval<br>• Include an approval number if one was obtained<br>• If the study involved *non-human primates*, add *additional details* about animal welfare and steps taken to ameliorate suffering<br>• If anesthesia, euthanasia, or any kind of animal sacrifice is part of the study, include briefly which substances and/or methods were applied<br><br>**Field Research**<br><br>Include the following details if this study involves the collection of plant, animal, or other materials from a natural setting:<br>• Field permit number<br>• Name of the institution or relevant body that granted permission	
**Data Availability**<br><br>Authors are required to make all data underlying the findings described fully available, without restriction, and from the time of publication. PLOS allows rare exceptions to address legal and ethical concerns. See the PLOS Data Policy and FAQ for detailed information.	Yes - all data are fully available without restriction

A Data Availability Statement describing where the data can be found is required at submission. Your answers to this question constitute the Data Availability Statement and **will be published in the article**, if accepted.<br><br>**Important:** Stating 'data available on request from the author' is not sufficient. If your data are only available upon request, select 'No' for the first question and explain your exceptional situation in the text box.<br><br>Do the authors confirm that all data underlying the findings described in their manuscript are fully available without restriction?	
**Describe where the data may be found in full sentences. If you are copying our sample text, replace any instances of XXX with the appropriate details.**<br><br>• If the data are **held or will be held in a public repository**, include URLs, accession numbers or DOIs. If this information will only be available after acceptance, indicate this by ticking the box below. For example: *All XXX files are available from the XXX database (accession number(s) XXX, XXX.).*<br>• If the data are all contained **within the manuscript and/or Supporting Information files**, enter the following: *All relevant data are within the manuscript and its Supporting Information files.*<br>• If neither of these applies but you are able to provide **details of access elsewhere**, with or without limitations, please do so. For example:<br><br>*Data cannot be shared publicly because of [XXX]. Data are available from the XXX Institutional Data Access / Ethics Committee (contact via XXX) for researchers who meet the criteria for access to confidential data.*<br><br>*The data underlying the results presented in the study are available from (include the name of the third party*	All relevant data are within the manuscript and its Supporting Information files.

and contact information or URL).<br>• This text is appropriate if the data are owned by a third party and authors do not have permission to share the data.<br><br>* typeset	
Additional data availability information:	

[29 June, 2019]

PLOS ONE

Dear Editor:

I wish to submit an original research article for publication in *PLOS ONE*, titled "Perceived discrimination and contextual problems among children and adolescents in Northern Chile." The paper was coauthored by Caqueo-Urízar A, Quintana L, Urzúa A, Irarrázaval M.

This study had two aims: first, to establish the effects of the relation between perceived discrimination and contextual problems (family, school and peers) in inter-ethnic Chilean students aged between 8 to 19 years from 29 schools in Arica, Chile; and second, to identify the role that ethnic identity and collective self-esteem play within this relation. This is an important fact because the cities in the northern region of Chile are subjects to many diverse and multicultural contexts, and previous studies have shown that migrant and minority populations are usually victims of discriminatory behavior. We believe that our study makes a significant contribution to the literature because it established that perceived discrimination and ethnic identity has direct effects over contextual problems, and collective self-esteem had a mediating role, so these constructs can be used to create strategized interventions to help diminish discriminatory behavior within schools in the Northern region of Chile and other applicable contexts.

Further, we believe that this paper will be of interest to the readership of your journal because teachers, school owners/managers and professionals related to children and adolescent development can utilize our findings to diminish discrimination in the school environment.

Please consider, Laura Zamarian as potential editor.

Thank you for your consideration. I look forward to hearing from you.

Sincerely,
Jerome Flores
Centro de Justicia Educacional, Universidad de Tarapacá
Avenida 18 de Septiembre 2222. Arica, Chile.
56-58-2205622
jflores@uta.cl

Manuscript Click here to access/download;Manuscript;Manuscript.docx

Perceived discrimination and contextual problems among children and adolescents in Northern Chile

Jerome Flores [1*¶], Alejandra Caqueo-Urízar [2¶], Lirna Quintana [1&], Alfonso Urzúa [3&], Matías Irarrázaval [4&]

[1] Centro de Justicia Educacional y Escuela de Psicología y Filosofía, Universidad de Tarapacá, Arica, Arica y Parinacota, Chile.

[2] Instituto de Alta Investigación. Universidad de Tarapacá, Arica, Arica y Parinacota, Chile.

[3] Escuela de Psicología, Universidad Católica del Norte, Antofagasta, Antofagasta, Chile.

[4] Departamento de Psiquiatría, Facultad de Medicina, Hospital Clínico Universidad de Chile, Santiago, Región Metropolitana, Chile.

* Corresponding author:

Email: jflores@uta.cl (J.F.)

[¶] These authors contributed equally to this work

[&] These authors also contributed equally to this work

Abstract

Discriminatory behaviors among inter-ethnical relations in schools have long been noted and studied, but there are several correlations between discriminatory behaviors and other constructs that need further investigation. As an example, the relation between perceived discrimination and contextual problems—which include family, school and peer problems—among children and adolescents in Latin America has received little attention from previous studies. Further, the mediating role of ethnic identity and collective self-esteem in this relation also needs to be considered as they could be proven as protective factors for discriminatory behavior and its outcomes. Therefore, this study aimed to, first, establish the effects of the relation between perceived discrimination and contextual problems in inter-ethnic students aged 8-19 years living in Arica, Chile; and second, to identify the role that ethnic identity and collective self-esteem play within this relation. In order to investigate this matter, a cross-sectional study was carried out with 3700 students in 29 schools between the fourth year of primary education and the last year of secondary education, aged between 9-18 years, with 48.4% men and 51.6% women. The sample was divided into primary and secondary groups. The scales utilized were the Everyday Discrimination Scale, Multi-Group Ethnic Identity Measure-Revised Scale, Collective Self-Esteem Scale and the dimensions of contextual family, school and peer problems, as well as the general index of contextual problems of the Child and Adolescent Assessment System. For data analysis, four models of structural equations were proposed to account for the relations between variables, two in each group, and all were optimally adjusted. We found that perceived discrimination and ethnic identity had a direct effect on contextual problems in general (.38-.45) and when separated (.29-.39), and collective self-esteem had only a mediating role. This study showed that strategized interventions focusing on ethnic identity and perceived discrimination should be utilized by schools to create a better developing environment.

Key words: perceived discrimination, ethnic identity, collective self-esteem, children and adolescents.

Introduction

It has been reported that migration to Chile has been increasing since 2015, with 14.8% of it being concentrated in the three northernmost regions of the country [1]. Further, according to data gathered from the Centro de Estudios del Ministerio de Educación de Chile [2], the number of foreign students has increased, and enrolment of foreign students is concentrated in the three northernmost regions, with 35% of the national total in 2015, and 30.7% in 2017.

As a result of this immigrational process, the schools within bordering cities face the constant challenge of including students from various nations into their educational system [3], and this is in consonance with the rights of the child determined by the United Nations, as it states that access to education should be guaranteed to children all around the world [4]. Therefore, in the three northernmost regions of Chile, over 80% of schools have foreign students enrolled [2], and this situation already poses a very complex problem for schools to handle as they have to deal with numerous cultural and ethnical differences within the same schooling environment, but this fact may become even more complex when taken into account that there are also native populations (from border cities) also enrolled in these schools, such as the Aymara in northern Chile [5].

Deepening this discussion, previous studies have shown that both migrant and ethnic minority populations have long been victims of discrimination [6, 7], and a study performed in Chile addressing inter-ethnic relations established that perceived discrimination plays a key role in how these relations develop [8]. This is not a surprise, as the Arica and Parinacota regions of Chile are subjects of many diverse and multicultural contexts, and the development of these relations is almost inevitable.

Regarding the discriminatory process, it has been noted that the experience of discrimination can affect mental and physical health in multiple ways during childhood and adolescence [9-11], and is a predictor of antisocial behavior and substance use [12]. However, previous studies have paid little attention to discrimination in the infantile-juvenile population in Chile, and some of the existing studies have solely focused on racial discrimination related to the Mapuche population and the Peruvian migrants [13, 14].

Furthermore, when considering the previous statements, it is not difficult to imagine that there may be students suffering daily with discrimination within schools in the northernmost region of Chile, and so it is possible to infer that there are constant tensions being generated in their relations with family, school, and peer since discrimination may affect children's perception of various aspects of their environment. It has been shown that discrimination affects family conflict, which in turn can mediate the relation between discrimination, well-being, and mental health in different populations [15-19]. Further, a negative correlation has been found between discrimination and students' perceived value/utility of their schools [20].

In addition, in Bronfenbrenner's ecological theory, three constructs were created to better conceptualize a child's environmental relations: the child's immediate environment includes his/her family and school, which constitute parts of the child's environmental microsystem; the interactions between these two microsystems comprise parts of the mesosystem, and the child's social values and various cultural aspects constitute parts of the macrosystem. A key aspect of this theory is the notion that the child is an active subject that is constantly interacting with his/her environment through progressive changes and adaptations, and that these changes and adaptations may vary in scope and form [21, 22].

Coinciding with this conceptualization, in a review study that analyzed the influences that the schooling cultural context may have on adolescents' development, it was concluded that most researches consistently demonstrate that adolescents actively construct their identities through social interaction; that these social interactions, in turn, are defined by the environments they inhabit; that these environments are either chosen by them or they are allowed to participate in; and, ultimately, that these constructed identities are critical to all aspects of their development [23]. Additionally, a review study on adolescent development in Latin America highlighted that the role of school and peer relations is a fundamental element in understanding children's emotional development and in addressing their ethnicity, and this may happen because there may be experiences of discrimination and exclusion within these relations that have been made invisible to others [24].

From an ecological-transactional perspective, a study demonstrated that attention should be directed to the child's family, school, and peer relation, so as to better understand how discrimination can affect the child's perceptions over these relations, and to better investigate the contextual problems that permeate them [25]. In terms of conceptualization, contextual problems refer to conflicts in the three main environmental relations that children and adolescents experience during their development: family problems, school problems, and peer problems. Further, it is also necessary to clarify the relation between perceived discrimination and contextual problems as a better understanding of this relation could be turned into strategized interventions to help mitigate discriminatory behavior.

Previous studies have also taken efforts to better conceptualize the relation between discrimination and well-being, and at least two mediators have been proposed: collective self-esteem and ethnic identity [15, 26]. In concept, collective self-esteem refers to an individual's own assessment and perceptions regarding others' assessments of the group to

which that individual belongs [27]. Regarding this construct, a previous study found that collective self-esteem has a mediating effect between perceived discrimination and emotional well-being in adolescents [26]. Moreover, the findings of another study showed that perceived discrimination has a negative effect on collective self-esteem [28], a fact that requires further investigation to ensure the validity of the correlation.

In concept, ethnic identity is defined as "that part of an individual's self-concept that derives from his or her knowledge of belonging to a social group, along with the evaluative and emotional meaning associated with that belonging" [29]. Additionally, this belonging can be linked to different social groups, including nationality, ethnicity, religion, among others. According to previous research, this mediator can be a protective factor against the negative consequences of many types of discrimination [27, 30, 31, 32].

However, it may not have the same effect on different ethnicities since some findings show that it may have a significant effect on African American's mental health, but not on Latin Americans [33]. It has also been observed that the lack of ethnic identity can have a detrimental effect, exacerbating somatic symptoms in the face of high cultural and educational stress [34]. In addition, in Chile, ethnic identity has been negatively correlated with the presence of antisocial behaviors [35], and the results obtained in another study suggest that ethnic identification is key to the maintenance of the Mapuche culture [36]. Additionally, it has been found that ethnic identity can predict collective self-esteem [37].

Based on these inferences and previous knowledge, we believe there is a need for further investigation to establish whether the two variables, ethnic identity, and collective self-esteem, can mediate the relationship between perceived discrimination and contextual problems. This is factual because they have been observed as mediators in other close relations - such as between discrimination and psychological well-being - and because both

seem to be directly linked to children' s/adolescents' identities. Thus, they are regarded as potential protective factors for discrimination.

In summary, there is a lack of studies in the relation between perceived discrimination and contextual problems in Latin America, especially in the infantile-juvenile population, and the role that collective self-esteem and ethnic identity may play within this relation is also unknown. Based on the aforementioned facts, we believe this study could contribute significantly to a better understanding of the effects of discrimination in schools within the northernmost regions of Chile and other applicable contexts.

Finally, the aim of this study was to, first, establish the effects of the relation between perceived discrimination and contextual problems in inter-ethnic students aged 8 to 19 living in the northern region of Chile; and second, to identify the role that ethnic identity and collective self-esteem play within this relation. Three hypotheses are posed from the objective: 1) The greater the perceived discrimination, the greater the contextual problems both in general and specifically. 2) Ethnic identity has a mediating role between perceived discrimination and contextual problems, both in general and specifically. 3) Collective self-esteem has a mediating role between perceived discrimination and contextual problems, both in general and specifically. Furthermore, age and sex were controlled in these relationships

Methodology

This is a non-experimental study because variables were not manipulated, and we used a cross-sectional correlational-causal approach, as we measured all variables in a single analysis.

Participants

The initial sample of this study included 3923 students from 29 schools situated in Arica, Chile, between the fourth year of primary education and the last year of secondary education, and the age range of these students was from 8 to 19 years. The sampling was done for convenience. It is a sample of the general school population, and there is no information on I.Q. or diagnosis of previous mental disorders. The SENA instrument has different versions of its instruments according to the age of the participants. While the contextual problem scales in both share the wording of most of their items (77%), varying the rest to suit age, and the secondary version has 1 item more than the primary version. It was then considered more accurate to divide the sample according to the version being used and to analyzed the two groups separately.

The division of the sample into primary and secondary groups is made on the basis of the grades in which the students are found, with the 8-12 years old version applying to fourth, fifth and sixth grades of primary and the 12-18 years old version applying to seventh until to last year of secondary. Originally the range of primary is from 8 to 11 years, but finally, the eight years old students were excluded as there were only 22 cases. Although 12-year-old students may answer the primary instrument, they were excluded from the sample to avoid confusion with 12-year-old students who answered the secondary version. There were no exclusion criteria other than the approval of the students' parents or guardians to participate, as well as the students' own willingness to participate.

To determine the final sample, we removed 1.8% of the students because they left more than 25% of the instruments unanswered, and it was used multiple imputation (MI) for missing values on the rest of the sample. Table 1 shows the baseline sociodemographic

statistics of the sample. The final total sample consisted of 3700 students, 1388 belonging to primary and 3212 to secondary schools, respectively. Where 48.4% were men, and the other 51.6% were women. In the primary group, the mean age was 10 years, and the standard deviation of 0.8. In the secondary group, the mean age was 14.4, and the standard deviation of 1.8. Through data analysis, we found that 91.9% of the students were Chilean, 3,7% Bolivian, 2.8% Peruvian, and the rest were from other nationalities. The ethnic groups with which the students identified themselves were: 54.4% Latin American, 25.9% Aymara, 5,2% Mapuche, 3% Afro-descendant, and the rest were distributed in other ethnic groups. The distribution by grade/course of the schools was quite homogeneous. This study included 39,8% of public schools, 55,5% of subsidized schools, and 4,7% of private schools.

**Table 1. Sociodemographic Characteristics of Participants at Baseline**		
Variable	n	%
Gender		
Female	1908	51.6
Male	1792	48.4
Grade		
4	532	14.4
5	469	12.7
6	387	10.5
7	501	13.5
8	460	12.4
9	407	11.0
10	358	9.7
11	325	8.8
12	261	7.1
Ethnicity		
Aymara	958	25.9
Quechua	38	1.0
Mapuche	192	5.2
Afro-descendant	110	3.0
Latin American	2011	54.4
Other	391	10.6

Nationality		
Chile	3401	91.9
Perú	103	2.8
Colombia	18	0.5
Venezuela	13	0.4
Bolivia	136	3.7
Ecuador	5	0.1
Argentina	4	0.1
Other	20	0.5
Type of school		
Public	1473	39.8
subsidized	2054	55.5
Private	173	4.7

Instruments

Everyday Discrimination Scale (EDS) [38]

A self-report instrument with an amount of evidence of internal consistency, and one-dimensional structure that measures discrimination in everyday life. This scale asks how many times subjects have felt discriminated against in their lives. It is a Likert scale consisted of 6 alternatives ranging from 1 (never) to 6 (almost every day). An example of writing is: "You were treated with less respect than other people." It has acceptable construct validity and adequate evidence of reliability, varying the alpha between 0.8 and 0.9. It was used the Spanish version of this instrument, which had previously been translated into Spanish by the original authors, and has been used in Latino adolescents in other studies in the U.S. [39]. The omega value was 0.88, both in primary and secondary school, in the present study. There is validation in Chile for university students, but not in adolescents. Data analysis was performed with the notion that a higher score meant a higher perceived discrimination.

Collective Self-Esteem Scale [27]

This instrument consists of 16 items grouped into four subscales, and each subscale consists of four items that are supposed to measure an individual's collective self-esteem. It is a self-report instrument. The four subscales are Membership Esteem, which evaluates the satisfaction with the roles that the individual occupies in the endo-group (i.e., immigrant group); Private Self-esteem, which evaluates the feeling of achievement and satisfaction that the individual obtains from his/her social belonging to the endo-group; Public Self-esteem, which is linked to the social validation of belonging, that is, how the endo-group is envisioned by the exo-group (i.e., society in general); and Importance to Identity, which is related to the level of importance that the endo-group places on the individual's self-concept. It is a Likert scale ranging from 1 (totally disagree) to 7 (totally agree). Data analysis was performed with the notion that a higher score meant higher collective self-esteem. This scale presented adequate evidence of reliability, with an alpha score of 0.76. In the present study, only Private Self-esteem and Importance to Identity dimensions were used, as it was regarded that the other two dimensions are not essential for this study's purposes since the questions were adjusted in relation to nationality. The Spanish version of this instrument was used, which had previously been translated in another study performed in Chile [40], but it has no validation in the infant and juvenile population. The omega value was 0.71 in the primary group, and 0.88 in the secondary group, in the present study.

Contextual Sub-scales of family, school, and peer problems from Child and Adolescent Assessment System (SENA) [41]

Developed by Spanish specialists in psychopathology and psychological evaluation, its purpose is to help in the detection of a wide range of infantile-juvenile emotional and

behavioral problems, and it is supposed to be used with children and adolescents ranging from 3 to 18 years old. It is a Likert scale and the answers to each item vary from 1 to 5 in the response options of each item, from "never or almost never" to "always or almost always". There are different versions of this instrument, according to whether the informant is the family, the school, or the student himself, as is the case in the present study. The total of each dimension is the average of the answers that constitute it and can vary between 1 and 5 continuously. It is worth noting that it has been built and validated entirely in Spanish. The results that can be obtained through its application are:

- Internalized problems: Depression, Anxiety, Social Anxiety, Somatic Complaints, Post-traumatic Symptomatology, and Obsession-Compulsion.
- Externalized Problems: Attention Problems, Anger Control Problems, Aggressive, Defiant, Hyperactive, and Antisocial behavior.
- Contextual problems: Family problems, School problems, and Peer problems.
- Specific Problems: Substance Use, Eating Behavior Problems, Developmental Delay, Learning Problems, Schizotypal Disorder, or Unusual Behavior.

Recently, a study has found that the Cronbach reliability of their subscales is above 0.7 in Spain [42]. In the current research, the three subscales of contextual problems have been used, as well as the general scale of contextual problems, composed of the three subscales. In the 8-12 age version, the sub-scale for family problems has 8 items, the sub-scale for school problems has 7 items, and the sub-scale for peer problems has 7 items, with a total of 22 items measuring contextual problems. In the 12-18 age version, the sub-scale of peer problems has one more item, with the number in the other two sub-scales remaining the same, being 23 items in a total of contextual problems in this version. An example of the

wording, in both versions, is: "I have problems at home" (Family problems). SENA has no validation in the infant and juvenile population in Chile. In the present study, the omega values were 0.78 for family problems, 0.76 for school problems, and 0.81 for peer problems in the primary group (8-12 age version). In the secondary group (12-18 age version), the omega values were 0.79 for family problems, 0.73 for school problems, and 0.75 for peer problems

Multigroup Ethnic Identity Measure-Revised (MEIM-R) [43]

This self-report instrument is supposed to provide an adequate measure of an individual's ethnic identity. Its revised version consists of 6 items that are scored on a Likert scale ranging from 1 (Totally disagree) to 5 (Totally agree). It has two dimensions: Exploration and Commitment. Previous studies have highlighted that instruments based on MEIM, in all its versions, are those that yield more significant results because it relates ethnic identity with other outcome variables, such as well-being or symptomatology [44]. An example of wording is: "I have a strong sense of belonging to my own ethnic group." The omega value was 0.88 in the primary group and 0.92 for the secondary group in the present study. It has validation in the infant and juvenile population in Chile [45].

Procedure

1. Approval of the ethics committee of the University of Tarapacá. This study is part of a larger Educational Justice Center project.
2. A total of 42 schools in the city of Arica were invited to participate in the study. In total, 29 schools (69%) agreed to participate in the study.

3. It was agreed with the principals of each school to have a 5-minute space at the regular parent-teacher meetings to invite them to participate in the study. Parents who did not attend the meetings received the invitation to participate through a communication from the headteachers of each course.
4. The study's aims and implications were duly explained to each parent, and they were then asked to give their written informed consent. Subsequently, the students themselves were provided with explanations regarding the study's aims and implications and then asked to give their written informed consent.
5. Each questionnaire was applied and finished within 45 minutes, and at least two trained interviewers were present in each room to answer questions regarding its content. At least one teacher of the respective establishment also accompanied the application in each classroom.

Data analysis

First, analyses were made regarding the sociodemographic descriptions of the sample, and the basic statistics of each variable in both groups. Then Pearson's correlations were calculated for principal variables in each group to establish a significative relationship between variables according to the first hypothesis and partially for the others hypothesis since a significant relationship is needed before analyzing more complex relationships. A multivariate analysis of variance (MANOVA) was conducted to assess the inclusion of gender and age variables in the subsequent structural equation models. Furthermore, structural equation modeling analysis (SEM) was performed in both groups to explore the mediating role of collective self-esteem and ethnic identity between discriminations and

contextual problems considering contextual problems as a unitary construct. Finally, SEM was performed again in both groups separating the contextual problems into three constructs: family, school, and peer. When analyzing goodness-of-fit, by convention, an RMSEA under .08 is acceptable, and under .05 is optimal, while a CFI and TLI higher than .9 are acceptable, and greater than .95 are optimal. SRMR is good below 0.8. According to previous studies, the CMIN (χ2/gl) is sensitive to sample sizes that go over 200 subjects/participants, although scores under 5 are considered acceptable even with classic criteria [46, 47]. Robust maximum likelihood (MLR) it was the estimator used in SEM, since it is robust even with not-normal distribution.

In this study, data were analyzed using the SPSS version 21.0 and the Mplus version 7.4. G*Power 3.1.9.7 was used to calculated size effect in MANOVA.Cohen [48] suggest the reference values for F^2: 0.02 small effect, 0.15 medium effect, and 0.35 large effect.

Little's MCAR test was used in both groups to analyzed missing data. In both groups, primary and secondary, it was identified a missing not at random data (MNAR). The items from everyday discrimination scale concentrate missing values with a range to 11.5 to 17.7% per item in primary, and 10.1 to15.4% in secondary. It was not found patterns with variables outside the everyday discrimination scale. Multiple imputation (MI) it was used to treatment of missing values; seven imputed data sets were generated in each group sample. In MI the goodness of fit is calculated in each imputed group, and the final result of each index is the average of all groups. This procedure does not allow for confidence intervals, but it does give the standard deviation.

Out-layers were identified in both groups, considering a z-score equal or greater than ± 3. In primary school, 89 cases (6,4%) were found, and 138 cases (6%) in secondary school.

These were not removed from the sample, but the difference between including them or not in the analyses is reported in the result section.

Results

Descriptive statistics and correlations

The asymmetry and kurtosis of both groups are presented in Table 2. It can be seen that the variable with the greatest asymmetry is peer problems. According to Ryu [49] it is acceptable up to 2 for the asymmetry and 7 for the kurtosis to consider using without problems the analysis that assumed the normal distribution.

Table 2. Asymmetry and Kurtosis

	Primary			Secondary	
Variable	Asymmetry	Kurtosis		Asymmetry	Kurtosis
ACOLECT	-.898	1.085		-1.003	.593
DISCRIM	1.286	.822		1.175	1.099
ID_ETNIC	-.045	-.918		.367	-.729
CONTEXT	1.522	2.568		1.164	1.663
P_FAMIL	1.661	3.087		1.092	.977
P_ESCUE	1.339	1.703		1.076	1.046
P_COMPA	2.061	5.049		2.376	7.443

Legend: ACOLECT = collective self-esteem. DISCRIM = perceived discrimination. ID_ETNIC = ethnic identity. CONTEXT = contextual problems. P_FAMIL = family problems. P_ESCUE = school problems. P_COMPA = peer problems.

Furthermore, Table 3 details the values of the means, standard deviations, and correlations of all variables in the study in both groups. It is observed that perceived discrimination, ethnic identity, and collective self-esteem correlate both with the separate

indices of contextual problems and also with contextual problems' general index. The exceptions in both groups are the non-significant correlations between ethnic identity and peer problems, and between ethnic identity and perceived discrimination, while its correlation with the overall index of contextual problems is the lowest of those that are significant.

Table 3. Pearson's correlations. means. and standard deviations of study variables

Primary		ACOLECT	DISCRIM	ID_ETNIC	CONTEXT	P_FAMIL	P_ESCUE	P_COMPA	*M*	*SD*
	ACOLECT	1							32.3	6.2
	DISCRIM	.243**	1						18.5	11.1
	ID_ETNIC	.092**	0.038	1					16.9	6.8
	CONTEXT	-.157**	-.426**	-.070**	1				52.6	10.2
	P_FAMIL	-.128**	-.347**	-.057*	.859**	1			1.6	0.6
	P_ESCUE	-.152**	-.283**	-.087**	.739**	.469**	1		1.7	0.7
	P_COMPA	-.100**	-.378**	-0.031	.754**	.444**	.382**	1	1.5	0.6
Secondary										
	ACOLECT	1							33.9	6
	DISCRIM	-.199**	1						18.8	9.2
	ID_ETNIC	.156**	0.007	1					14.3	6.4
	CONTEXT	-.149**	.446**	-.086**	1				51.4	8.6
	P_FAMIL	-.155**	.312**	-.066**	.768**	1			1.7	0.7
	P_ESCUE	-.113**	.314**	-.156**	.729**	.373**	1		1.8	0.7
	P_COMPA	-.077**	.397**	0.007	.760**	.315**	.387**	1	1.3	0.5

Legend: ACOLECT = collective self-esteem. DISCRIM = perceived discrimination. ID_ETNIC = ethnic identity. CONTEXT = contextual problems. P_FAMIL = family problems. P_ESCUE = school problems. P_COMPA = peer problems.

* $p < 0.05$ (bilateral). ** $p < 0.01$ (bilateral).

Multivariate analysis of variance

A multivariate analysis of variance (MANOVA) was conducted considering gender and age as independent variables in both groups. The dependent variables were collective self-esteem. discrimination. ethnic identity. contextual problems. problems with the family. problems with school and problems with peer. Tables 4 and 5 show the significance of the main effects, and interaction, and the marginal means, respectively, for both groups. The box test is significant in both groups; therefore, the Pillai trace was considered in the global models.

In the primary group, the Pillai trace value was significant for the main effects. being found for sex of F(7.1394) =7.685. p< .001. f^2=.07. and for age of F(14.2790) =3.403. p< .001. f^2=.02. No significant effect was found on the interaction between gender and age being F(42.13710) =1.516. p=.212. f^2=.003. Boys presented greater perceived discrimination, contextual problems in general, as well as problems with school and peer. In ethnic identity the age is significant. being found in the DMS post-hoc tests that the 11-year-old group is different from the other two.

In secondary, the Pillai Trace Value was significant for the main effects. being found a value for gender of F(7.2280) =6.610. p< .001. f^2=.044. and for age of F(42.13710) =5.529. p< .001. f^2=.01. A significant effect was also found for the interaction between gender and age. F(42.13710) =1.516. p< .05. f^2=.001.

The gender differences are in collective self-esteem, perceived discrimination, ethnic identity, general contextual problems, school problems, and peer problems, with ethnic identity being the only one greater in girls, and the rest are all greater in boys. With respect to age, significant differences were found in collective self-esteem. Post-hoc Gabriel tests

found that the 12-year-old group differed significantly in collective self-esteem from the 14. 15. 16 and 17-year-old groups, *p*<.000. The 13-year old group differs from the 15-year old group, *p*<.05, and the 17-year old group, *p*<.000, decreasing in all of them as age increases. An interaction effect between gender and age was obtained. *p*<.05, finding that at 16 years of age, the highest value of collective self-esteem is produced in boys and the lowest in girls.

Table 4. Resume of significant effects

	**Primary**			**Secondary**		
Variable	Gender	Age	Gender * Age	Gender	Age	Gender * Age
ACOLECT	.062	.325	.910	.020*	.000**	.015*
EDST	.000**	.661	.398	.003**	.209	.893
ID_ETNIC	.239	.012**	.058	.028*	.753	.310
PD_CTX	.000**	.409	.104	.006**	.350	.166
PD_FAM	.058	.526	.100	.067	.287	.494
PD_ESC	.000**	.113	.018**	.000**	.492	.935
PD_COM	.012**	.267	.785	.000**	.569	.080

Legend: ACOLECT = collective self-esteem. DISCRIM = perceived discrimination. ID_ETNIC = ethnic identity. CONTEXT = contextual problems. P_FAMIL = family problems. P_ESCUE = school problems. P_COMPA = peer problems.

* p < 0.05 (bilateral). ** p < 0.01 (bilateral).

Note: interaction effect in primary it was not explained since the global model was not significant.

Table 5. Marginal Means of MANOVA

	Primary		Secondary	
	Gender	Age	Gender	Age

Variable	Boys	Girls	9	10	11		Boys	Girls	12	13	14	15	16	17	18
ACOLECT	31.9	32.8	32.5	32.0	32.8		34.3	33.5	35.7	34.9	33.6	33.1	33.5	32.2	34.3
DISCRIM	20.8	16.6	17.8	19.2	18.4		19.7	17.8	17.8	18.7	19.1	19.6	19.2	18.7	16.9
ID_ETNIC	16.6	17.2	17.3	17.5	15.8		14.0	15.0	15.1	14.4	14.4	14.3	14.3	14.2	14.9
CONTEXT	53.0	50.2	50.7	51.3	52.3		51.5	50.1	50.9	51.3	50.7	51.0	51.0	50.6	48.0
P_FAMIL	1.5	1.5	1.5	1.5	1.5		1.7	1.7	1.7	1.7	1.7	1.7	1.7	1.7	1.5
P_ESCUE	1.8	1.5	1.6	1.6	1.7		1.9	1.7	1.7	1.8	1.8	1.8	1.8	1.8	1.7
P_COMPA	1.5	1.4	1.3	1.4	1.4		1.4	1.3	1.4	1.3	1.3	1.3	1.4	1.3	1.2

Legend: ACOLECT = collective self-esteem. DISCRIM = perceived discrimination. ID_ETNIC = ethnic identity. CONTEXT = contextual problems. P_FAMIL = family problems. P_ESCUE = school problems. P_COMPA = peer problems.

Analysis of the unitary model of contextual problems

Unitary SEM in the primary group

Fig 1 shows the unitary model of contextual problems in the primary group. It can be seen that ethnic identity does not show a mediation effect since it is not affected by perceived discrimination. At the same time, collective self-esteem does appear as a mediator between perceived discrimination and contextual problems, as well as between ethnic identity and contextual problems. Ethnic identity does have a direct effect on collective self-esteem (r = .160; p<.001); Perceived discrimination does have a direct effect on contextual problems (r = .377; p<.001); and a negative direct effect on collective self-esteem (r = -.203; p<.001); there is a significant indirect effect of ethnic identity on contextual problems (r = -0.020; p<.005); and a significant indirect effect of perceived discrimination on contextual problems (r = 0.025; p<.005). Age presented a direct effect on collective self-esteem (r = .057; p<.001); Gender presented a direct effect on contextual problems (r = .067; p<.001), finding that being a boy predicts greater problems. Finally, collective self-esteem presents a negative direct

effect on contextual problems (*r* = -.124; *p*<.001). Regarding this analysis. goodness-of-fit indicators are optimal. as demonstrated herein: $\chi2$/gl = 2.13. CFI = .981. TLI = .977. RMSEA = 0.028 (*SD* = .001), AIC = 62393.516, BIC = 62634.355, SRMR = 0.035.

Figure 1. Primary SEM considering contextual problems as a unitary construct with their standardized values. Legend: Identic = ethnic identity. Discri = perceived discrimination. Aucole = collective self-esteem. Pd_pctx = Contextual Problems.

Unitary SEM in the secondary group

Fig 2 shows the unitary model of contextual problems in the secondary group. It can be seen that ethnic identity does not show a mediation effect since it is not affected by perceived discrimination, but it shows a negative direct effect on contextual problems(*r* = -.096; *p*<.05); there is also a significant negative indirect effect of ethnic identity on contextual problems (*r* = -0.011; *p*<.05). Further, ethnic identity presented a direct effect on collective self-esteem (*r* = .196; *p*<.001). At the same time, collective self-esteem does appear as a mediator between perceived discrimination and contextual problems, as well as between ethnic identity and contextual problems. Furthermore, perceived discrimination does have a direct effect on contextual problems (*r* = .453; *p*<.001); and a significant indirect effect on the same variable (*r* = 0.010; *p*<.01). Perceived discrimination also presented a negative direct effect on collective self-esteem (*r* = -.167; *p*<.001). Collective self-esteem presented a negative direct effect on contextual problems (*r* = -.058; *p*<.01); Age presented a negative direct effect on collective self-esteem (*r* = -.179; *p*<.001), and a negative direct effect on

contextual problems (*r* = -.071; *p*<.01); Gender presented a direct effect on collective self-esteem (*r* = .064; *p*<.001), finding that being a boy predicts better collective self-esteem. Regarding this analysis, goodness-of-fit indicators are optimal, as demonstrated herein: χ2/gl = 4.66, CFI = .973, TLI = .967, RMSEA = 0.040 (*SD* = .001), AIC = 94034.088, BIC = 94304.326, SRMR = 0.031.

Figure 2. Secondary SEM considering contextual problems as a unitary construct with their standardized values. Legend: Identic = ethnic identity. Discri = perceived discrimination. Aucole = collective self-esteem. Pd_pctx = Contextual Problems.

Divided model analysis of contextual problems

Divided SEM in the primary group

Fig 3 shows the standardized values of the contextual problem model separated by family, school, and peer. In this analysis, ethnic identity is observed to have a negative direct effect on school problems (*r* = -.064; *p*<.05), and it also has a negative indirect effect, mediated by collective self-esteem, on family problems (*r* = -.016; *p*<.01), and on school problems (*r* = -.019; *p*<.01), and peer problems (*r* = -.013; *p*<.01). Ethnic identity does have a direct effect on collective self-esteem (*r* = .159; *p*<.001). Further, perceived discrimination has a direct effect on the three types of contextual problems, being greater in peer problems (*r* = .328; *p*<.001). followed by family problems (*r* = .292; *p*<.001), and by school problems (*r* = .256; *p*<.001). Therefore, perceived discrimination also presented a negative direct effect on collective self-esteem (*r* = -.198; *p*<.001). In turn, there is an indirect effect of perceived

discrimination on family problems (*r* = .020; *p*<.01), and on school problems (*r* = .024; *p*<.01), and on peer problems (*r* = .016; *p*<.05). Collective self-esteem is observed to have a negative direct effect in all specific contextual problems, being greater in school problems (*r* = -.120; *p*<.001), followed by family problems (*r* = -.102; *p*<.01), and by peer problems (*r* = -.083; *p*<.01). Age shows a direct effect on school problems (*r* = .090; *p*<.001), and collective self-esteem (*r* = .057; *p*<.05). Gender presented a direct effect in school problems (*r* = .120; *p*<.001), finding that being a boy predicts greater problems. Regarding this analysis, goodness-of-fit indicators are optimal, as demonstrated herein: $\chi 2$/gl = 2.17, CFI = .980, TLI = .972, RMSEA = .029 (*SD* = .001), AIC = 59326.853, BIC = 59667.169, SRMR = 0.032.

Figure 3. Primary SEM considering contextual problems as separate constructs. with their standardized values. Legend: Identic = ethnic identity. Discri = perceived discrimination. Aucole = collective self-esteem. Pd_famil = family problems. Pd_escue = school problems. Pd_compa = peer problems.

Divided SEM in the secondary group

Fig 4 shows the standardized values of the contextual problem model separated by family, school, and peer. In this analysis, ethnic identity is observed to have a negative direct effect on family problems (*r* = -.070; *p*<.01), and school problems (*r* = -.161; *p*<.001), and it also has a negative indirect effect, mediated by collective self-esteem, on family problems (*r* = -.013; *p*<.01). Ethnic identity does have a direct effect on collective self-esteem (*r* = .196; *p*<.001). Further, perceived discrimination has a direct effect on the three types of contextual

problems, being greater in peer problems (*r* = .389; *p*<.001). followed by school problems (*r* = .347; *p*<.001), and by family problems (*r* = .301; *p*<.001). Therefore, perceived discrimination also presented a negative direct effect on collective self-esteem (*r* = -.167; *p*<.001). In turn, there is an indirect effect of perceived discrimination family problems (*r* = .011; *p*<.01). Collective self-esteem is observed to have a negative direct effect on family problems (*r* = -.069; *p*<.01). Age shows a negative direct effect on peer problems (*r* = -.059; *p*<.001). and collective self-esteem (*r* = -.179; *p*<.001). Gender presented a direct effect in all specific contextual problems, being greater in peer problems (*r* = .102; *p*<.001), followed by a negative effect on family problems (*r* = -.095; *p*<.001), and by school problems (r = .066; p<.001). Finding that being a boy predicts greater peer and school problems, and being a girl predicts greater family problems. Regarding this analysis, goodness-of-fit indicators are optimal. as demonstrated herein: $\chi 2$/gl = 4.85. CFI = .968, TLI = .958, RMSEA = .041 (*SD* = .001), AIC = 89266.287, BIC = 89628.521, SRMR = 0.030.

Figure 4. Secondary SEM considering contextual problems as separate constructs. with their standardized values. Legend: Identic = ethnic identity. Discri = perceived discrimination. Aucole = collective self-esteem. Pd_famil = family problems. Pd_escue = school problems. Pd_compa = peer problems.

Goodness-of-fit for all models are optimal since all parameters are beyond the acceptable ranges, and some even beyond the optimal ranges. Finally, collective self-esteem was regarded with a role of partial mediation for both ethnic identity and perceived discrimination in all models.

Effect to removes out-layers

The results of removing the out-layers before multiple imputation should be considered as reference. In both primary and secondary groups, the unitary model, all the effects in relationships are maintained, and the maximum reduction of the effects reaches .03.

In the specific primary model, all relationships are maintained, except for the effect of ethnic identity on school problems, which is no longer significant. A gender relationship appears on peer problems (.06). The maximum effect variation in all relationships is around .03, except for the effect of perceived discrimination on school problems, which decreases by .07. In the specific model for secondary schools, all relationships are maintained, and the maximum variation in the effects on relationships is .04. In all these SEM models, optimum goodness fit indicators are maintained.

Discussions

The aim of this study was divided into two sections: first. to establish the effects of the relation between perceived discrimination and contextual problems in inter-ethnic students aged 9 to 19 living in the northern region of Chile; and second, to identify the role that ethnic identity and collective self-esteem play within this relation. The three hypotheses that derived from this objective were: 1) The greater the perceived discrimination, the greater the contextual problems both in general and specifically. 2) Ethnic identity has a mediating role between perceived discrimination and contextual problems, both in general and specifically. 3) Collective self-esteem has a mediating role between perceived discrimination and contextual problems, both in general and specifically.

Relation between perceived discrimination and contextual problems

It was found evidence that fully supports the first hypothesis in both groups. The results indicated that perceived discrimination did have an effect on contextual problems, whether considered as a whole or separately, so it can be inferred that exacerbated discriminatory behaviors towards students may increase their family, school, and peer problems. Further, when considered separately, it can be noted that peer problems was the most affected area by perceived discrimination. In opposition to this finding, early studies on the ecological model gave more importance to students' school relations with their teachers/school than with their peer [21. 22]. However, since our findings demonstrate that peer problems is the most affected area when students experience discriminatory behaviors, it seems necessary to give this type of relation at least an equally important role to that of teachers/school when theorizing the importance of school relations for students. Further, our results coincide with recent research on children and adolescent's development, which consider peer relations equally as important as family and teachers/school relations [24. 25].

In addition, there are newer propositions regarding the ecological model which stated that humans develop through progressively more complex reciprocal interactions, which involve an individual (in this case. the student) and the objects, symbols and individuals in his/her external environment (in this case. family. school and peer), and that, for these interactions to effectively impact the individual's development. these interactions must be regular and placed over extended periods of time; this new concept was called proximal process [50]. Thus, based on the concepts of proximal processes, if we consider that peer spend much more time with the student than their parents or teachers/school, it is quite logical to assume that their role is much more relevant than what this same theory originally stated

and, therefore, it seems relevant to pay attention to the effects of perceived discrimination on peer problems when analyzing children and adolescent development.

Moreover, peer relations and problems are generally studied within the perspective of bullying [51]. However, this is a limitation in this study because there are other conflicts that do not necessarily involve students being bullied and still involve discriminatory behaviors. Thus, further investigations on these relations are needed since they are relevant for children's and adolescents' development and may even be central in that regard.

Now. when taking into account the direct effect of perceived discrimination on family problems, we found that it is consistent with the results obtained by previous research [11. 19], although they used different instruments to measure family problems. These findings are also in line with the results obtained by a study conducted with an adult Latin American population [18].

As for school problems, a review study noted that previous studies have usually investigated the student-teacher relationship in the context of teacher discriminatory behavior or in aspects not directly related to conflict, such as school climate investigations; and that previous studies have usually investigated the direct student-school relation in the contexts of size, culture, and safety of the school for the students [23]. Thus, since this study's findings indicate that school problems are also affected by discrimination. it seems necessary for further studies to investigate school problems, as it may further improve the understanding of children's and adolescents' development.

It should be noted that in unitary models, the direct effect of perceived discrimination on contextual problems increases by .07 in the secondary group. Likewise, in divided models the effect of discrimination on school and peer problems increases by .06 or more, but only .01 in family problems. It is possible to hypothesize that secondary school students have

experienced a cumulative effect of perceived discrimination, and this increases the direct effect of discrimination on contextual problems. Future research may address this possibility.

Ethnic identity and collective self-esteem role.

The second hypothesis was not supported by evidence since ethnic identity was not affected by perceived discrimination in any group. Furthermore, in the unitary models of both groups, ethnic identity neither had no significant effect on contextual problems. In the divided models of both groups, it was not related to all specific contextual problems, but to some. The present results rule out the mediation of ethnic identity in the relation between perceived discrimination and contextual problems, although it may have presented a direct effect on some contextual problems in the divided model's analysis. In both groups, ethnic identity has a direct effect on school problems, but only in the secondary group also affect to family problems. These results are supported by research which stated that perceived discrimination is not significantly related to the ethnic identity of adolescents, although they may have used different instruments to measure discrimination [52, 53].

However, one of these previous studies has brought up another variable into this question: maybe ethnic identity has not matured enough in adolescents, so it would not establish such a relation with discrimination due to it still being in development [52]. In partial corroboration to that notion, another study performed on Latin American adults was able to effectively find a relation between both variables (ethnic identity and perceived discrimination) [54]. On the other hand, another study performed on Asian American adults was not able to effectively find a relation between both variables [55].

Notwithstanding, some of these results partially contradict what a previous study found using the same MEIM-R scale that was used in this study, as a relation between

perceived discrimination and ethnic identity in adolescents was found [56]. In addition, other studies that did not necessarily use this scale have also found solid relations between both variables in adolescents [28. 31. 57]. In short, there are mixed results that do not allow to either discard or affirm this relation, and this has been previously highlighted in a previous meta-analysis study [44].

Furthermore, the direct effect of ethnic identity on family problems found in this research contradicts the findings of previous research, which stated that ethnic identity significantly affected parent-child relations in African Americans, but not in Latin Americans [33].

Evidence was found that partially supports the third hypothesis of the study, which affirms the collective self-esteem mediation relationship between discrimination and contextual problems. In unitary models of both groups, mediation appears, but in divided models, it does not appear completely, because collective self-esteem affects all contextual problems only in the primary school group. In the secondary group, it affects exclusively family problems.

Moreover, the negative effect of discrimination on collective self-esteem is expected and is consistent with previous research conducted with adolescents [26. 28], as well as those conducted with children [58]. However, in another study, a relation between the two variables was not found in children, but a different instrument was used to measure discrimination, and that should be taken into account when comparing both [59].

Additionally, the relation between ethnic identity and collective self-esteem coincides with the results of a previous study which found a positive relation between the two [28] and another study that found that ethnic identity predicts collective self-esteem [37]. Furthermore, it is logical to situate both variables within the macrosystem of

Bronfenbrenner's theory since they form part of the culture in which the individual is immersed.

Further, this research had the opportunity of including latent variables, which implies that we did consider the statistical measurement error through an adequate treatment of missing values – by multiple imputation (MI) in the present study - taking into account the recommendations of previous studies [60]. This same study emphasized that significant statistical effects should not be discarded, even if they were modest, but it is quite clear that the role of mediation of collective self-esteem does not seem sufficient to focus strategized interventions on it. On the other hand, perceived discrimination and ethnic identity seemed as more reasonable focal points when creating strategized intervention efforts, and since both ethnic identity and collective self-esteem are linked to the individual's identity, it seems reasonable to include the measurement of identity when strategizing these interventions.

In terms of strengths, the models of structural equations proposed in this study had appropriate goodness-of-fit indicators, and specifics models provided relevant findings for this study's aim. Another strength was the wide age range of participants, and this is a relevant aspect because this type of study usually investigates either adolescents or children, not both.

Nonetheless, this study had several limitations. In terms of data gathering, convenience sampling was performed, so it reduced the possibilities of result generalization, and further studies should try to select more specific sample groups to provide more accurate notions regarding the topic of study. Further, only one region of the country was considered and incorporated, so further studies that encompass a more geographically varied sample would be ideal. Moreover, although the SEM models were guided and performed based on previous theories, they did not offer experimental evidence that allows for the establishment of causality among the variables. Future studies should try to utilize statistical models that

may allow for the establishment of causality among the variables to further improve the range of conclusions of the study. Finally, this study also relies exclusively on student's self-reports, so other studies that offer different types of reports and observations in terms of data collection are required to further investigate perceived discrimination among children and adolescents in Chile. Furthermore, although the SENA instrument has an inconsistency scale, it does not have a social desirability scale.

Finally, regarding future researches, we believe that there are some specific changes regarding the methodological approach that could further contribute to the understanding of the studied phenomenon, and are described herein: including other contextual aspects and problems, such as the children's and adolescents' neighborhood; the inclusion of other variables, such as personal self-esteem in the statistical model; the inclusion of others' perspectives regarding the discriminatory process, such as the parents' and teachers' (an action that would be consistent with the ecological model that was explored in this study); and changing the study's perspective to a longitudinal one.

Conclusions

In conclusion, this study showed that perceived discrimination affects contextual problems - whether considered as a whole or separately - at the family, school and peer levels; ethnic identity has a direct effect on school problems in primary and secondary students, and in this last group also in family problems; and collective self-esteem has a mediating role for both ethnic identity and perceived discrimination over contextual problems.

Acknowledgements

This research has been funded by ANID PIA CIE160007.

References

1. Instituto Nacional de Estadísticas. Departamento de Demografía y Censos (2018) Características de la inmigración internacional en Chile. Censo 2017. Available from: http://www.censo2017.cl/descargas/inmigracion/181123-documento-migracion.pdf
2. Ministerio de Educación. Centro de Estudios (2018). Mapa del estudiantado extranjero en el sistema escolar chileno (2015-2017). Documento de trabajo N° 12. Santiago. Chile. Available from: https://www.mineduc.cl/wp-content/uploads/sites/19/2018/05/MAPA_ESTUDIANTES_EXTRANJEROS_SISTEMA_ESCOLAR_CHILENO_2015_2017.pdf
3. de Aquino RIC. Wiesner MLR. García AAE. Moreno AS. Educación inclusiva y niñez migrante: barreras a la educación de niñez de origen centroamericano en escuelas de Tapachula. Chiapas (México). Revista de Investigación en Educación. 2016;14(1): 85-104.
4. United Nations General Assembly. Convention on the Rights of the Child. [cited 11 June 2019] United Nations. Treaty Series. 1989;1577: 3. Available from: https://www.refworld.org/docid/3ae6b38f0.html.
5. Guizardi M. Las fronteras del transnacionalismo. 1st ed. Santiago de Chile: Ocho libros; 2015.
6. Hopenhayn M. Bello A. Discriminación étnico-racial y xenofobia en América Latina y el Caribe. Santiago de Chile: CEPAL; 2001.

7. Neblett EW. Rivas- Drake D. Umaña- Taylor AJ. The promise of racial and ethnic protective factors in promoting ethnic minority youth development. Child Devel Perspec. 2012;6(3): 295-303. doi: 10.1111/j.1750-8606.2012.00239.x.
8. Tropp. L.R. O'Brien TC. Gutierrez RG. Valdenegro D. Migacheva K. de Tezanos-Pinto P. et al. How school norms. peer norms. and discrimination predict interethnic experiences among ethnic minority and majority youth. Child Devel. 2016;87(5): 1436-1451. doi: 10.1111/cdev.12608.
9. Busse D. Yim IS. Campos B. Social context matters: Ethnicity. discrimination and stress reactivity. Psychoneuroendocrinology. 2017;83: 187–193. doi: 10.1016/j.psyneuen.2017.05.025.
10. Pachter LM. Caldwell CH. Jackson JS. Bernstein BA. Discrimination and mental health in a representative sample of African-American and Afro-Caribbean youth. J Racial Ethnic Health Disp. 2017;5(4): 831-837. doi: 10.1007/s40615-017-0428-z.
11. Ríos-Salas V. Larson A. Perceived discrimination. socioeconomic status. and mental health among Latino adolescents in U.S. immigrant families. Children and Youth Serv Rev. 2015;56: 116-125. doi: 10.1016/j.childyouth.2015.07.011.
12. Walsh SD. Kolobov T. Simanovskaya O. What is it about perceived discrimination that can lead immigrant adolescents to alcohol use and delinquency? The mediating role of feelings of alienation. Subst Use Misuse. 2019;54(1): 65-77. doi: 10.1080/10826084.2018.1495738.
13. Becerra S. Merino M. Mellor D. Ethnic discrimination against Mapuche students in urban high schools in the Araucanía region. Chile. Inter Educ Stud. 2015;8(10). doi: 10.5539/ies.v8n10p96.

14. Lahoz S. Forns M. Discriminación percibida. afrontamiento y salud mental en migrantes peruanos en Santiago de Chile. Psicoperspectivas. 2015;15(1): 157-168. doi: 10.5027/PSICOPERSPECTIVAS-VOL15-ISSUE1-.
15. Cheng HL. Lin SP. Cha CH. Perceived discrimination. intergenerational family conflicts. and depressive symptoms in foreign-born and U.S.-born Asian American emerging adults. Asian American J Psychol. 2015;6(2): 107-116. doi: 10.1037/a0038710.
16. Juang L.P. Alvarez AA. Discrimination and adjustment among Chinese American adolescents: Family conflict and family cohesion as vulnerability and protective factors. Am J Public Health. 2010;100(12): 2403-2409. doi: 10.2105/AJPH.2009.185959.
17. Juang L.P. Alvarez AA. Family. school. and neighborhood: Links to Chinese American adolescent perceptions of racial/ethnic discrimination. Asian Am J Psychol. 2011;2(1): 1-12. doi: 10.1037/a0023107.
18. Molina KM. Little T.V. Rosal MC. Everyday discrimination. family context. and psychological distress among Latino adults in the United States. J Comm Psychol. 2016;44(2): 145-165. doi: 10.1002/jcop.21747.
19. Ponting C. Steve SS. Escovar E.L. Rappa AM. Camacho A. Calderon I. et al. Family factors mediate discrimination related stress and externalizing symptoms in rural Latino adolescents. J Adolesc. 2018;69: 11-21. doi: 10.1016/j.adolescence.2018.08.007.
20. Wong CA. Eccles JS. Sameroff A. The influence of ethnic discrimination and ethnic identification on African American adolescents' school and socioemotional adjustment. J Pers. 2003;71(6): 1197-1232. doi: 10.1111/1467-6494.7106012

21. Bronfenbrenner U. The ecology of human development. Cambridge: Harvard University Press; 1979.
22. Bronfenbrenner U. Discovering what families do. In: Blankenhorn D. Bayme S. Elshtain J.B. Rebuilding the nest: A new commitment to the American family. Milwaukee: Family Service America; 1990. pp. 27-38.
23. Eccles JS. Roeser RW. Schools as developmental contexts during adolescence. J Res Adolesc. 2011;21(1): 225–241. doi: 10.1111/j.1532-7795.2010.00725.x.
24. Preiss DD. Calcagni E. Grau V. Classroom research and child and adolescent development in South America. New Dir Child Adolesc Dev. 2015;2015(147): 85-92. In: Jensen LA. Larson RW. The global context for new directions for child and adolescent development PREFACE. New Dir Child Adolesc Dev. 2015;147: 1-2. doi: 10.1002/cad.20090.
25. Hughes DL. Watford JA. Del Toro J. A transactional/ecological perspective on ethnic–racial identity. socialization. and discrimination. Adv Child Dev Behav. 2016;51: 1-41. In: Horn S. Ruck M. Liben L. Advances in child development and behavior. Volume 51: Equity and justice in developmental science: Implications for young people. families. and communities. 1st ed. San Diego: Elsevier Academic Press; 2016. Pp. 1-41.
26. Jia X. Liu X. Shi B. Perceived discrimination and subjective well-being in Chinese migrant adolescents: Collective and personal self-esteem as mediators. Front Psychol. 2017;8: 1213. doi: 10.3389/fpsyg.2017.01213.
27. Luhtanen R. Crocker J. A Collective Self-Esteem Scale: Self-evaluation of one's social identity. Pers Social Psychol Bull. 1992;18(3): 302-318.

28. Liu X. Zhao J. Chinese migrant adolescents' perceived discrimination and psychological well-being: The moderating roles of group identity and the type of school. PLOS ONE. 2016;11(1): e0146559. doi: 10.1371/journal.pone.0146559.

29. Tajfel H. Grupos humanos y categorías sociales. Barcelona: Herder; 1984.

30. Cavanaugh AM. Stein GL. Supple AJ. Gonzalez LM. Kiang L. Protective and promotive effects of Latino early adolescents' cultural assets against multiple types of discrimination. J Res Adolesc. 2018;28(2): 310-326. doi: 10.1111/jora.12331.

31. Marcelo AK. Yates TM. Young children's ethnic–racial identity moderates the impact of early discrimination experiences on child behavior problems. Cultur Divers Ethnic Minor Psychol. 2019;25(2): 253-265. doi: 10.1037/cdp0000220.

32. Romero AJ. Edwards LM. Fryberg SA. Orduña M. Resilience to discrimination stress across ethnic identity stages of development. J App Soc Psychol. 2013;44(1): 1-11. doi: 10.1111/jasp.12192.

33. Hurwich- Reiss E. Rienks SL. Bianco H. Wadsworth ME. Markman HJ. Exploring the role of ethnic identity in family functioning among low- income parents. J Comm Psychol. 2015;43(5): 545–559. doi: 10.1002/jcop.21701.

34. Torres SA. Santiago CD. Culture and educational stress and internalizing symptoms among Latino adolescents: The role of ethnic identity. J Edu Psychol Consult. 2017;27(3): 344–366. doi: 10.1080/10474412.2017.1301819.

35. Jiménez-Bustos RA. Arenas RXPL. Rivera GEB. Identidad étnica y conductas sociales en adolescentes indígenas Mapuche sancionados por la Ley de Responsabilidad Penal Adolescente en regiones del sur de Chile. Univ Psychol. 2017;16(1): 1-22. doi: 10.11144/Javeriana.upsy16-1.iecs.

36. González R. Lickel B. Gupta M. Tropp LR. Luengo Kanacri BP. Mora E. et al. Ethnic identity development and acculturation preferences among minority and majority youth: Norms and Contact. Child Dev. 2017;88(3): 743-760. doi: 10.1111/cdev.12788.
37. Jetten J. Branscombe N.R. Haslam SA. Haslam C. Cruwys T. Jones JM. et al. Having a lot of a good thing: Multiple important group memberships as a source of self-esteem. PLOS ONE 2015;10(5): e0131035. doi: 10.1371/journal.pone.0124609.
38. Williams DR. Yu Y. Jackson JS. Anderson NB. Racial differences in physical and mental health: Socioeconomic status. stress. and discrimination. J Health Psychol. 1997;2(3): 335-351. doi: 10.1177/135910539700200305.
39. Krieger N. Smith K. Naishadham D. Hartman C. Barbeau EM. Experiences of discrimination: validity and reliability of a self-report measure for population health research on racism and health. Soc Sci Med. 2005;61(7): 1576-1596.
40. Espinosa A. Acosta Y. Valencia J. Vera A. Soares da Silva A. Romero JC. et al. Calidez. competencia. moralidad y nacionalismo ideal como dimensiones autoestereotípicas del autoconcepto nacional en seis países de Latinoamérica. Avances en Psicología Latinoamericana. 2006;34(2): 395-413.
41. Fernández-Pinto I. Santamaría P. Sánchez-Sánchez F. Carrasco MA. del Barrio V. Sistema de evaluación de niños y adolescentes. Manual de aplicación. corrección e interpretación. Madrid: TEA Ediciones; 2015.
42. Sánchez-Sánchez F. Fernández-Pinto I. Santamaría P. Carrasco MA. del Barrio V. SENA. Sistema de Evaluación de Niños y Adolescentes: proceso de desarrollo y evidencias de fiabilidad y validez. Revista de Psicología Clínica con Niños y Adolescentes. 2016;3(2): 23-34.

43. Phinney JS. Ong AD. Conceptualization and measurement of ethnic identity: Current status and future directions. J Couns Psychol. 2007;54(3): 271-281. doi: 10.1037/0022-0167.54.3.271.

44. Rivas-Drake D. Syed M. Umaña-Taylor A. Markstrom C. French S. Schwartz SJ. et al. Feeling good. happy. and proud: A meta-analysis of positive ethnic-racial affect and adjustment. Child Dev. 2014;85(1): 77–102. doi:10.1111/cdev.12175.

45. L ara, L. & Martínez-Molina, A. (2016). Validación de la Escala de Identidad étnica Multigrupo-Revisada en adolescentes inmigrantes y autóctonos residentes en España. *Revista Latinoamericana de Ciencias Sociales, Niñez y Juventud*, *14* (1), pp. 591-601. http://dx.doi.org/10.11600/1692715x.14140110515

46. Brown TA. Methodology in the social sciences. Confirmatory factor analysis for applied research. 2nd ed. New York: The Guilford Press; 2015.

47. McDonald RP. Moon-Ho RH. Principles and practice in reporting structural equation analyses. Psychol Meth. 2002;7(1): 64-82. doi: 10.1037/1082-989X.7.1.64.

48. Cohen J. Statistical power analysis for the behavioral sciences. Abingdon. England: Routledge. 1988.

49. Ryu E. Effects of skewness and kurtosis on normal-theory based maximum likelihood test statistic in multilevel structural equation modeling. Behavior Research Methods, 2011;43(4), 1066-1074. doi: 10.3758/s13428-011-0115-7

50. Bronfenbrenner U. Morris PA. The bioecological model of human development. In: Lerner RM. Damon W. Handbook of child psychology: Theoretical models of human development. Hoboken: John Wiley & Sons Inc; 2006. pp. 793-828.

51. Lorenzo-Blanco EI. Unger JB. Oshri A. Baezconde-Garbanati L. Soto D. Profiles of bullying victimization. discrimination. social support. and school safety: Links with

Latino/a youth acculturation. gender. depressive symptoms. and cigarette use. Am J Orthopsychiatry. 2016;86(1): 37-48. doi: 10.1037/ort0000113.

52. Myrick SE. Martorell GA. Sticks and stones may break my bones: Protective factors for the effects of perceived discrimination on social competence in adolescence. Pers Rel. 2011;18(3): 487-501. doi: 10.1111/j.1475-6811.2010.01320.x.
53. Tummala-Narra P. Claudius M. Perceived discrimination and depressive symptoms among immigrant-origin adolescents. Cultur Divers Ethnic Minor Psychol. 2013;19(3): 257-269. doi: 10.1037/a0032960.
54. Molina KM. Estrella ML. Durazo-Arvizu R. Malcarne VL. Llabre MM. Isasi CR. et al. Perceived discrimination and physical health-related quality of life: The Hispanic Community Health Study/Study of Latinos (HCHS/SOL) Sociocultural Ancillary Study. Soc Sci Med. 2019;222: 91-100. doi: 10.1016/j.socscimed.2018.12.038.
55. Yoo H. Lee R. Ethnic identity and approach-type coping as moderators of the racial discrimination/well-being relation in Asian Americans. J Couns Psychol. 2005;52(4): 497-506. doi: 10.1037/0022-0167.52.4.497.
56. Cristini F. Scacchi L. Perkins D.D. Santinello M. Vieno A. The influence of discrimination on immigrant adolescents' depressive symptoms: What buffers its detrimental effects? Psychosoc Interv. 2011;20(3): 243-253. doi: 10.5093/in2011v20n3a2.
57. Romero AJ. Roberts RE. The impact of multiple dimensions of ethnic identity on discrimination and adolescents' self-esteems. J App Soc Psychol. 2003;33(11): 2288–2305. doi: 10.1111/j.1559-1816.2003.tb01885.x.

58. Cassidy C. O'Connor RC. Howe C. Warden D. Perceived discrimination and psychological distress: The role of personal and ethnic self-esteem. J Couns Psychol. 2004;51(3): 329–339. doi: 10.1037/0022-0167.51.3.329.

59. Lam BT. Impact of perceived racial discrimination and collective self-esteem on psychological distress among Vietnamese-American college students: sense of coherence as mediator. Am J Orthopsychiatry. 2007;77(3). 370–376. doi: 10.1037/0002-9432.77.3.370.

60. Wolf EJ. Harrington KM. Clark SL. Miller. M.W. Sample size requirements for structural equation models: An evaluation of power. bias. and solution propriety. Educ Psychol Meas. 2013;76(6): 913-934. doi: 10.1177/0013164413495237.

Figure4

Click here to access/download;Figure;Fig4.tif

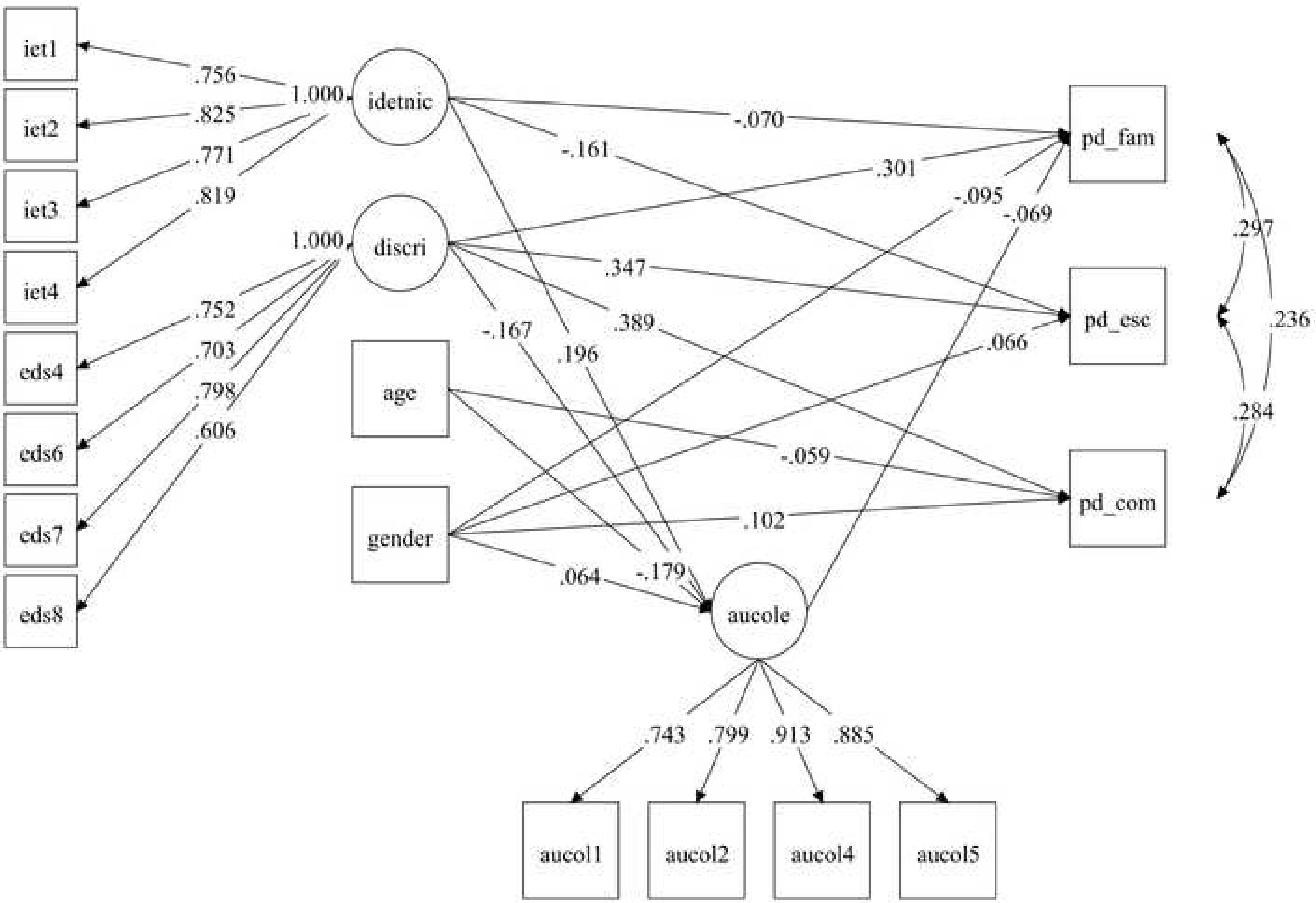

Figure1

Click here to access/download;Figure;Fig1.tif

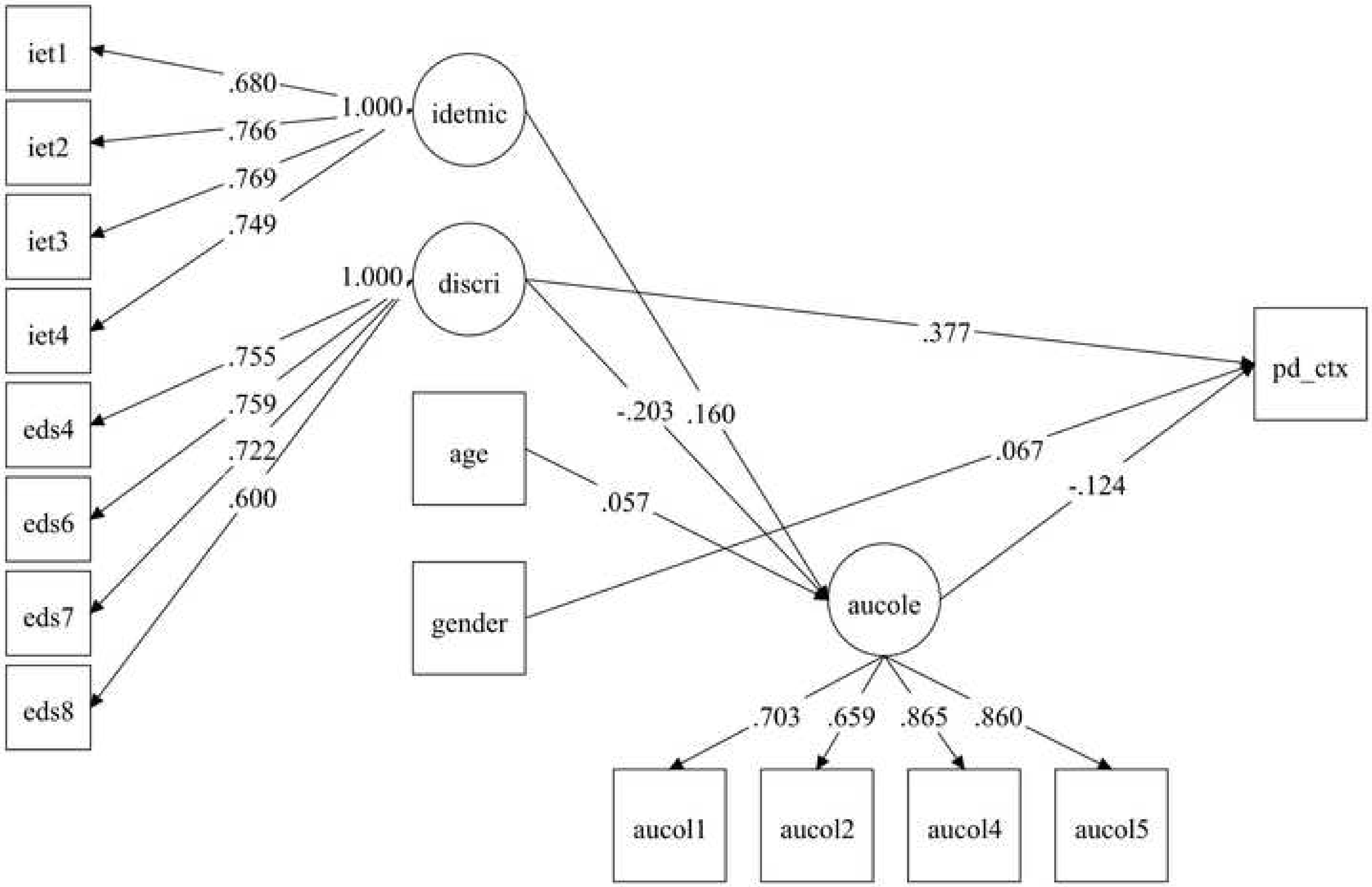

Figure2

Click here to access/download;Figure;Fig2.tif

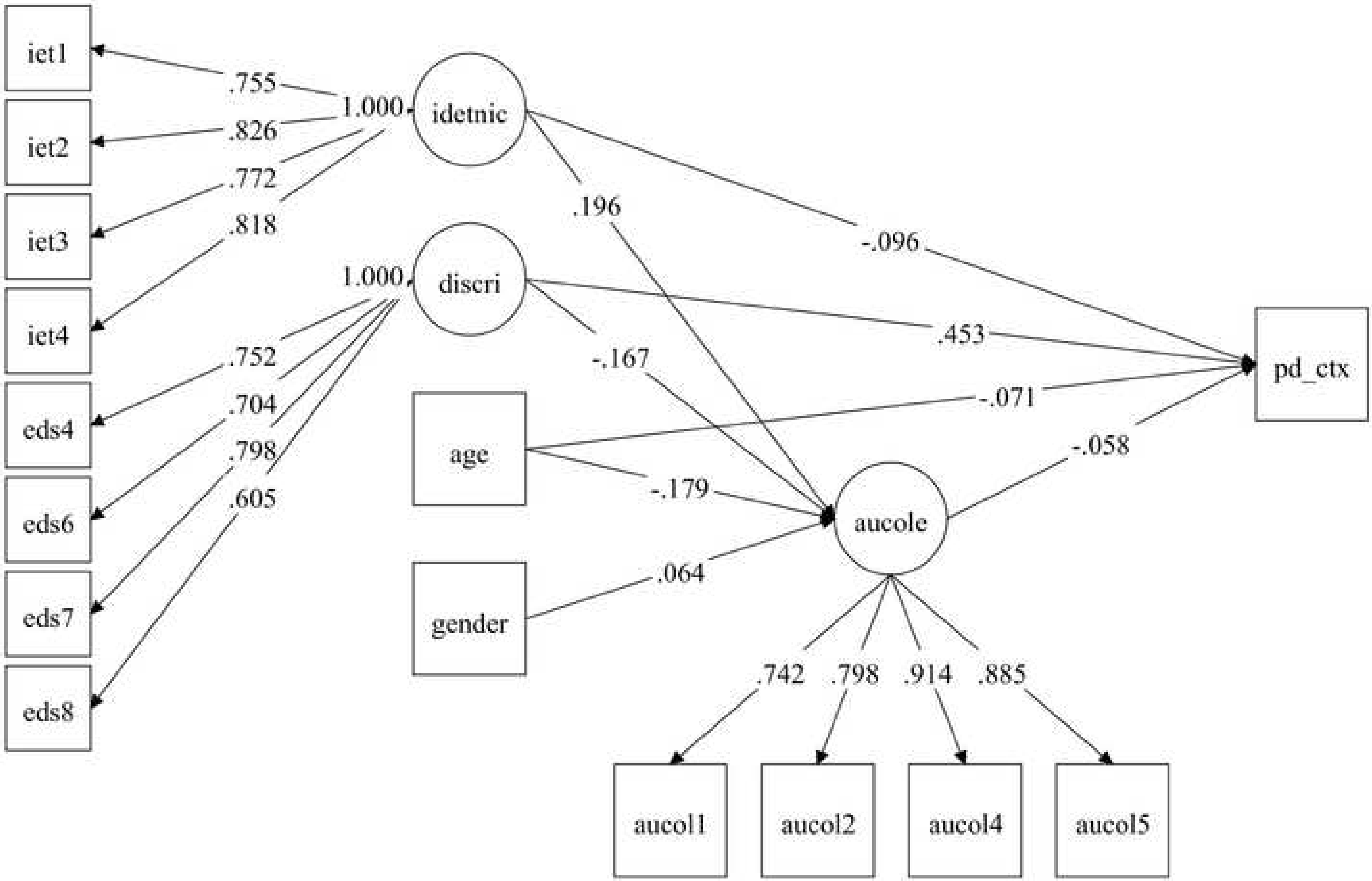

Figure3

Click here to access/download;Figure;Fig3.tif

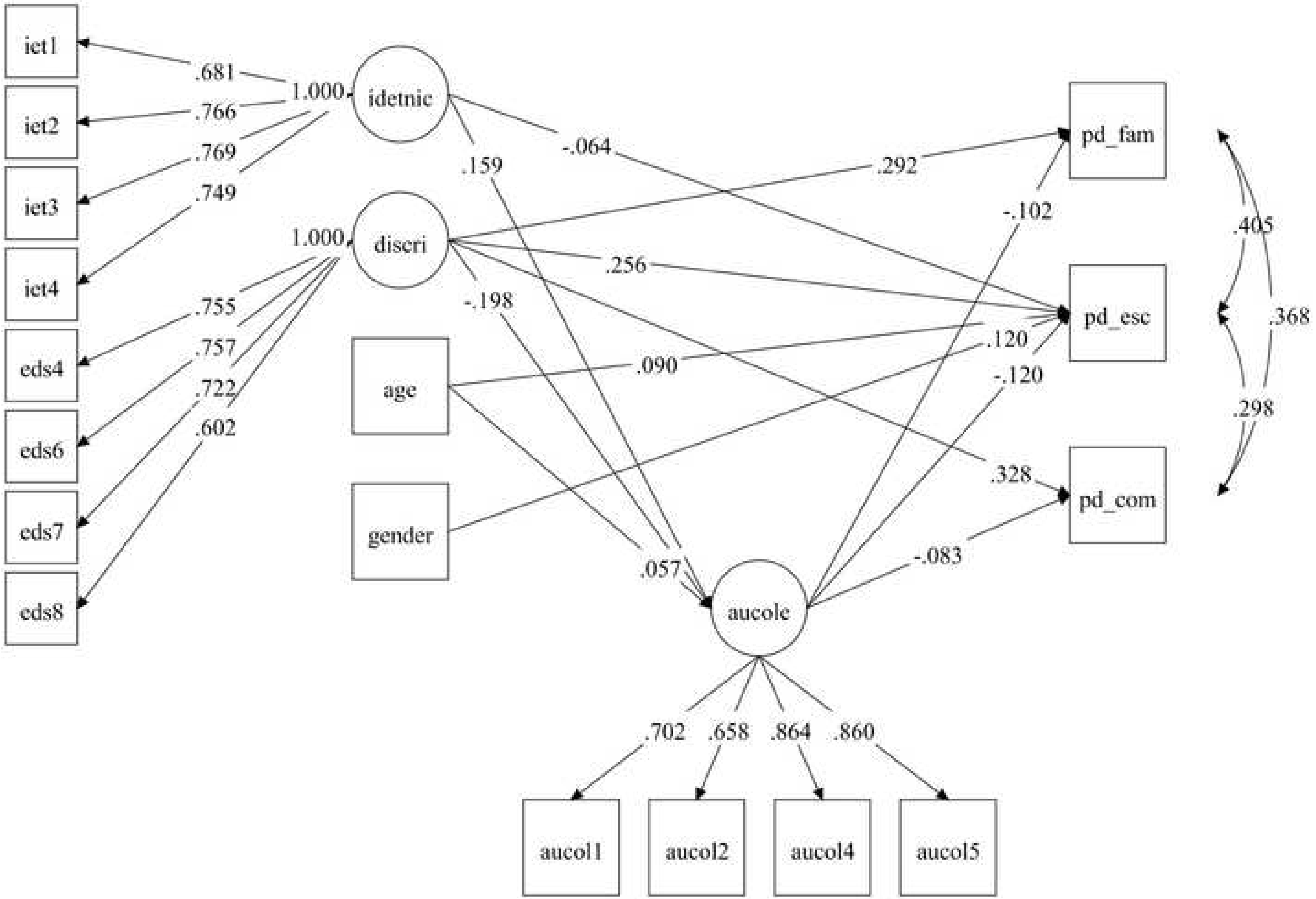

DATASET PRIMARY GROUP

Click here to access/download
Supporting Information
MATCH Primaria TOT PLOSONE3.sav

DATASET SECONDARY GROUP

Click here to access/download
Supporting Information
MATCH Secundaria TOT PLOSONE3.sav

SYNTAXIS FOR BOTH GROUPS

Click here to access/download

Supporting Information

SYNTAXIS MPLUS 8 PRIMARY AND SECONDARY.docx

Variable Codes

Click here to access/download
Supporting Information
code variables dataset plosone.xlsx

Click here to access/download
Other
Certificate_of_editing-ALURZ_8.pdf

Reply to editor

Click here to access/download
Other
Reply editor before second review.doc

Perceived discrimination and contextual problems among children and adolescents in Northern Chile

Jerome Flores [1*¶], Alejandra Caqueo-Urízar [2¶], Lirna Quintana [1&], Alfonso Urzúa [3&], Matías Irarrázaval [4&]

[1] Centro de Justicia Educacional y Escuela de Psicología y Filosofía, Universidad de Tarapacá, Arica, Arica y Parinacota, Chile.

[2] Centro de Justicia Educacional e Instituto de Alta Investigación. Universidad de Tarapacá, Arica, Arica y Parinacota, Chile.

[3] Escuela de Psicología, Universidad Católica del Norte, Antofagasta, Antofagasta, Chile.

[4] Departamento de Psiquiatría, Facultad de Medicina, Hospital Clínico Universidad de Chile, Santiago, Región Metropolitana, Chile.

* Corresponding author:

Email: jflores@uta.cl (J.F.)

[¶] These authors contributed equally to this work

[&] These authors also contributed equally to this work

Abstract

Discriminatory behaviors among inter-ethnical relations in schools have long been noted and studied, but there are several correlations between discriminatory behaviors and other constructs that need further investigation. As an example, the relation between perceived discrimination and contextual problems—which include family, school and peer problems—among children and adolescents in Latin America has received little attention from previous studies. Further, the mediating role of ethnic identity and collective self-esteem in this relation also needs to be considered as they could be proven as protective factors for discriminatory behavior and its outcomes. Therefore, this study aimed to, first, establish the effects of the relation between perceived discrimination and contextual problems in inter-ethnic students aged 8-19 years living in Arica, Chile; and second, to identify the role that ethnic identity and collective self-esteem play within this relation. In order to investigate this matter, a cross-sectional study was carried out with 3700 students in 29 schools between the fourth year of primary education and the last year of secondary education, aged between 9-18 years, with 48.4% men and 51.6% women. The sample was divided into primary and secondary groups. The scales utilized were the Everyday Discrimination Scale, Multi-Group Ethnic Identity Measure-Revised Scale, Collective Self-Esteem Scale and the dimensions of contextual family, school and peer problems, as well as the general index of contextual problems of the Child and Adolescent Assessment System. For data analysis, four models of structural equations were proposed to account for the relations between variables, two in each group, and all were optimally adjusted. We found that perceived discrimination and ethnic identity had a direct effect on contextual problems in general (.38-.45) and when separated (.29-.39), and collective self-esteem had only a mediating role. This study showed that strategized interventions focusing on ethnic identity and perceived discrimination should be utilized by schools to create a better developing environment.

Key words: perceived discrimination, ethnic identity, collective self-esteem, children and adolescents.

Introduction

It has been reported that migration to Chile has been increasing since 2015, with 14.8% of it being concentrated in the three northernmost regions of the country [1]. Further, according to data gathered from the Centro de Estudios del Ministerio de Educación de Chile [2], the number of foreign students has increased, and enrolment of foreign students is concentrated in the three northernmost regions, with 35% of the national total in 2015, and 30.7% in 2017.

As a result of this immigrational process, the schools within bordering cities face the constant challenge of including students from various nations into their educational system [3], and this is in consonance with the rights of the child determined by the United Nations, as it states that access to education should be guaranteed to children all around the world [4]. Therefore, in the three northernmost regions of Chile, over 80% of schools have foreign students enrolled [2], and this situation already poses a very complex problem for schools to handle as they have to deal with numerous cultural and ethnical differences within the same schooling environment, but this fact may become even more complex when taken into account that there are also native populations (from border cities) also enrolled in these schools, such as the Aymara in northern Chile [5].

Deepening this discussion, previous studies have shown that both migrant and ethnic minority populations have long been victims of discrimination [6, 7], and a study performed in Chile addressing inter-ethnic relations established that perceived discrimination plays a key role in how these relations develop [8]. This is not a surprise, as the Arica and Parinacota regions of Chile are subjects of many diverse and multicultural contexts, and the development of these relations is almost inevitable.

Regarding the discriminatory process, it has been noted that the experience of discrimination can affect mental and physical health in multiple ways during childhood and adolescence [9-11], and is a predictor of antisocial behavior and substance use [12]. However, previous studies have paid little attention to discrimination in the infantile-juvenile population in Chile, and some of the existing studies have solely focused on racial discrimination related to the Mapuche population and the Peruvian migrants [13, 14].

Furthermore, when considering the previous statements, it is not difficult to imagine that there may be students suffering daily with discrimination within schools in the northernmost region of Chile, and so it is possible to infer that there are constant tensions being generated in their relations with family, school, and peer since discrimination may affect children's perception of various aspects of their environment. It has been shown that discrimination affects family conflict, which in turn can mediate the relation between discrimination, well-being, and mental health in different populations [15-19]. Further, a negative correlation has been found between discrimination and students' perceived value/utility of their schools [20].

In addition, in Bronfenbrenner's ecological theory, three constructs were created to better conceptualize a child's environmental relations: the child's immediate environment includes his/her family and school, which constitute parts of the child's environmental microsystem; the interactions between these two microsystems comprise parts of the mesosystem, and the child's social values and various cultural aspects constitute parts of the macrosystem. A key aspect of this theory is the notion that the child is an active subject that is constantly interacting with his/her environment through progressive changes and adaptations, and that these changes and adaptations may vary in scope and form [21, 22].

Coinciding with this conceptualization, in a review study that analyzed the influences that the schooling cultural context may have on adolescents' development, it was concluded that most researches consistently demonstrate that adolescents actively construct their identities through social interaction; that these social interactions, in turn, are defined by the environments they inhabit; that these environments are either chosen by them or they are allowed to participate in; and, ultimately, that these constructed identities are critical to all aspects of their development [23]. Additionally, a review study on adolescent development in Latin America highlighted that the role of school and peer relations is a fundamental element in understanding children's emotional development and in addressing their ethnicity, and this may happen because there may be experiences of discrimination and exclusion within these relations that have been made invisible to others [24].

From an ecological-transactional perspective, a study demonstrated that attention should be directed to the child's family, school, and peer relation, so as to better understand how discrimination can affect the child's perceptions over these relations, and to better investigate the contextual problems that permeate them [25]. In terms of conceptualization, contextual problems refer to conflicts in the three main environmental relations that children and adolescents experience during their development: family problems, school problems, and peer problems. Further, it is also necessary to clarify the relation between perceived discrimination and contextual problems as a better understanding of this relation could be turned into strategized interventions to help mitigate discriminatory behavior.

Previous studies have also taken efforts to better conceptualize the relation between discrimination and well-being, and at least two mediators have been proposed: collective self-esteem and ethnic identity [15, 26]. In concept, collective self-esteem refers to an individual's own assessment and perceptions regarding others' assessments of the group to

which that individual belongs [27]. Regarding this construct, a previous study found that collective self-esteem has a mediating effect between perceived discrimination and emotional well-being in adolescents [26]. Moreover, the findings of another study showed that perceived discrimination has a negative effect on collective self-esteem [28], a fact that requires further investigation to ensure the validity of the correlation.

In concept, ethnic identity is defined as "that part of an individual's self-concept that derives from his or her knowledge of belonging to a social group, along with the evaluative and emotional meaning associated with that belonging" [29]. Additionally, this belonging can be linked to different social groups, including nationality, ethnicity, religion, among others. According to previous research, this mediator can be a protective factor against the negative consequences of many types of discrimination [27, 30, 31, 32].

However, it may not have the same effect on different ethnicities since some findings show that it may have a significant effect on African American's mental health, but not on Latin Americans [33]. It has also been observed that the lack of ethnic identity can have a detrimental effect, exacerbating somatic symptoms in the face of high cultural and educational stress [34]. In addition, in Chile, ethnic identity has been negatively correlated with the presence of antisocial behaviors [35], and the results obtained in another study suggest that ethnic identification is key to the maintenance of the Mapuche culture [36]. Additionally, it has been found that ethnic identity can predict collective self-esteem [37].

Based on these inferences and previous knowledge, we believe there is a need for further investigation to establish whether the two variables, ethnic identity, and collective self-esteem, can mediate the relationship between perceived discrimination and contextual problems. This is factual because they have been observed as mediators in other close relations - such as between discrimination and psychological well-being - and because both

seem to be directly linked to children' s/adolescents' identities. Thus, they are regarded as potential protective factors for discrimination.

In summary, there is a lack of studies in the relation between perceived discrimination and contextual problems in Latin America, especially in the infantile-juvenile population, and the role that collective self-esteem and ethnic identity may play within this relation is also unknown. Based on the aforementioned facts, we believe this study could contribute significantly to a better understanding of the effects of discrimination in schools within the northernmost regions of Chile and other applicable contexts.

Finally, the aim of this study was to, first, establish the effects of the relation between perceived discrimination and contextual problems in inter-ethnic students aged 8 to 19 living in the northern region of Chile; and second, to identify the role that ethnic identity and collective self-esteem play within this relation. Three hypotheses are posed from the objective: 1) The greater the perceived discrimination, the greater the contextual problems both in general and specifically. 2) Ethnic identity has a mediating role between perceived discrimination and contextual problems, both in general and specifically. 3) Collective self-esteem has a mediating role between perceived discrimination and contextual problems, both in general and specifically. Furthermore, age and sex were controlled in these relationships

Methodology

This is a non-experimental study because variables were not manipulated, and we used a cross-sectional correlational-causal approach, as we measured all variables in a single analysis.

Participants

The initial sample of this study included 3923 students from 29 schools situated in Arica, Chile, between the fourth year of primary education and the last year of secondary education, and the age range of these students was from 8 to 19 years. The sampling was done for convenience. It is a sample of the general school population, and there is no information on I.Q. or diagnosis of previous mental disorders. The SENA instrument has different versions of its instruments according to the age of the participants. While the contextual problem scales in both share the wording of most of their items (77%), varying the rest to suit age, and the secondary version has 1 item more than the primary version. It was then considered more accurate to divide the sample according to the version being used and to analyzed the two groups separately.

The division of the sample into primary and secondary groups is made on the basis of the grades in which the students are found, with the 8-12 years old version applying to fourth, fifth and sixth grades of primary and the 12-18 years old version applying to seventh until to last year of secondary. Originally the range of primary is from 8 to 11 years, but finally, the eight years old students were excluded as there were only 22 cases. Although 12-year-old students may answer the primary instrument, they were excluded from the sample to avoid confusion with 12-year-old students who answered the secondary version. There were no exclusion criteria other than the approval of the students' parents or guardians to participate, as well as the students' own willingness to participate.

To determine the final sample, we removed 1.8% of the students because they left more than 25% of the instruments unanswered, and it was used multiple imputation (MI) for missing values on the rest of the sample. Table 1 shows the baseline sociodemographic

statistics of the sample. The final total sample consisted of 3700 students, 1388 belonging to primary and 3212 to secondary schools, respectively. Where 48.4% were men, and the other 51.6% were women. In the primary group, the mean age was 10 years, and the standard deviation of 0.8. In the secondary group, the mean age was 14.4, and the standard deviation of 1.8. Through data analysis, we found that 91.9% of the students were Chilean, 3,7% Bolivian, 2.8% Peruvian, and the rest were from other nationalities. The ethnic groups with which the students identified themselves were: 54.4% Latin American, 25.9% Aymara, 5,2% Mapuche, 3% Afro-descendant, and the rest were distributed in other ethnic groups. The distribution by grade/course of the schools was quite homogeneous. This study included 39,8% of public schools, 55,5% of subsidized schools, and 4,7% of private schools.

Table 1. Sociodemographic Characteristics of Participants at Baseline

Variable	n	%
Gender		
Female	1908	51.6
Male	1792	48.4
Grade		
4	532	14.4
5	469	12.7
6	387	10.5
7	501	13.5
8	460	12.4
9	407	11.0
10	358	9.7
11	325	8.8
12	261	7.1
Ethnicity		
Aymara	958	25.9
Quechua	38	1.0
Mapuche	192	5.2
Afro-descendant	110	3.0
Latin American	2011	54.4
Other	391	10.6

Nationality		
Chile	3401	91.9
Perú	103	2.8
Colombia	18	0.5
Venezuela	13	0.4
Bolivia	136	3.7
Ecuador	5	0.1
Argentina	4	0.1
Other	20	0.5
Type of school		
Public	1473	39.8
subsidized	2054	55.5
Private	173	4.7

Instruments

Everyday Discrimination Scale (EDS) [38]

A self-report instrument with an amount of evidence of internal consistency, and one-dimensional structure that measures discrimination in everyday life. This scale asks how many times subjects have felt discriminated against in their lives. It is a Likert scale consisted of 6 alternatives ranging from 1 (never) to 6 (almost every day). An example of writing is: "You were treated with less respect than other people." It has acceptable construct validity and adequate evidence of reliability, varying the alpha between 0.8 and 0.9. It was used the Spanish version of this instrument, which had previously been translated into Spanish by the original authors, and has been used in Latino adolescents in other studies in the U.S. [39]. The omega value was 0.88, both in primary and secondary school, in the present study. There is validation in Chile for university students, but not in adolescents. Data analysis was performed with the notion that a higher score meant a higher perceived discrimination.

Collective Self-Esteem Scale [27]

This instrument consists of 16 items grouped into four subscales, and each subscale consists of four items that are supposed to measure an individual's collective self-esteem. It is a self-report instrument. The four subscales are Membership Esteem, which evaluates the satisfaction with the roles that the individual occupies in the endo-group (i.e., immigrant group); Private Self-esteem, which evaluates the feeling of achievement and satisfaction that the individual obtains from his/her social belonging to the endo-group; Public Self-esteem, which is linked to the social validation of belonging, that is, how the endo-group is envisioned by the exo-group (i.e., society in general); and Importance to Identity, which is related to the level of importance that the endo-group places on the individual's self-concept. It is a Likert scale ranging from 1 (totally disagree) to 7 (totally agree). Data analysis was performed with the notion that a higher score meant higher collective self-esteem. This scale presented adequate evidence of reliability, with an alpha score of 0.76. In the present study, only Private Self-esteem and Importance to Identity dimensions were used, as it was regarded that the other two dimensions are not essential for this study's purposes since the questions were adjusted in relation to nationality. The Spanish version of this instrument was used, which had previously been translated in another study performed in Chile [40], but it has no validation in the infant and juvenile population. The omega value was 0.71 in the primary group, and 0.88 in the secondary group, in the present study.

Contextual Sub-scales of family, school, and peer problems from Child and Adolescent Assessment System (SENA) [41]

Developed by Spanish specialists in psychopathology and psychological evaluation, its purpose is to help in the detection of a wide range of infantile-juvenile emotional and

behavioral problems, and it is supposed to be used with children and adolescents ranging from 3 to 18 years old. It is a Likert scale and the answers to each item vary from 1 to 5 in the response options of each item, from "never or almost never" to "always or almost always". There are different versions of this instrument, according to whether the informant is the family, the school, or the student himself, as is the case in the present study. The total of each dimension is the average of the answers that constitute it and can vary between 1 and 5 continuously. It is worth noting that it has been built and validated entirely in Spanish. The results that can be obtained through its application are:

- Internalized problems: Depression, Anxiety, Social Anxiety, Somatic Complaints, Post-traumatic Symptomatology, and Obsession-Compulsion.
- Externalized Problems: Attention Problems, Anger Control Problems, Aggressive, Defiant, Hyperactive, and Antisocial behavior.
- Contextual problems: Family problems, School problems, and Peer problems.
- Specific Problems: Substance Use, Eating Behavior Problems, Developmental Delay, Learning Problems, Schizotypal Disorder, or Unusual Behavior.

Recently, a study has found that the Cronbach reliability of their subscales is above 0.7 in Spain [42]. In the current research, the three subscales of contextual problems have been used, as well as the general scale of contextual problems, composed of the three subscales. In the 8-12 age version, the sub-scale for family problems has 8 items, the sub-scale for school problems has 7 items, and the sub-scale for peer problems has 7 items, with a total of 22 items measuring contextual problems. In the 12-18 age version, the sub-scale of peer problems has one more item, with the number in the other two sub-scales remaining the same, being 23 items in a total of contextual problems in this version. An example of the

wording, in both versions, is: "I have problems at home" (Family problems). SENA has no validation in the infant and juvenile population in Chile. In the present study, the omega values were 0.78 for family problems, 0.76 for school problems, and 0.81 for peer problems in the primary group (8-12 age version). In the secondary group (12-18 age version), the omega values were 0.79 for family problems, 0.73 for school problems, and 0.75 for peer problems

Multigroup Ethnic Identity Measure-Revised (MEIM-R) [43]

This self-report instrument is supposed to provide an adequate measure of an individual's ethnic identity. Its revised version consists of 6 items that are scored on a Likert scale ranging from 1 (Totally disagree) to 5 (Totally agree). It has two dimensions: Exploration and Commitment. Previous studies have highlighted that instruments based on MEIM, in all its versions, are those that yield more significant results because it relates ethnic identity with other outcome variables, such as well-being or symptomatology [44]. An example of wording is: "I have a strong sense of belonging to my own ethnic group." The omega value was 0.88 in the primary group and 0.92 for the secondary group in the present study. It has validation in the infant and juvenile population in Chile [45].

Procedure

1. Approval of the ethics committee of the University of Tarapacá. This study is part of a larger Educational Justice Center project.
2. A total of 42 schools in the city of Arica were invited to participate in the study. In total, 29 schools (69%) agreed to participate in the study.

3. It was agreed with the principals of each school to have a 5-minute space at the regular parent-teacher meetings to invite them to participate in the study. Parents who did not attend the meetings received the invitation to participate through a communication from the headteachers of each course.
4. The study's aims and implications were duly explained to each parent, and they were then asked to give their written informed consent. Subsequently, the students themselves were provided with explanations regarding the study's aims and implications and then asked to give their written informed consent.
5. Each questionnaire was applied and finished within 45 minutes, and at least two trained interviewers were present in each room to answer questions regarding its content. At least one teacher of the respective establishment also accompanied the application in each classroom.

Data analysis

First, analyses were made regarding the sociodemographic descriptions of the sample, and the basic statistics of each variable in both groups. Then Pearson's correlations were calculated for principal variables in each group to establish a significative relationship between variables according to the first hypothesis and partially for the others hypothesis since a significant relationship is needed before analyzing more complex relationships. A multivariate analysis of variance (MANOVA) was conducted to assess the inclusion of gender and age variables in the subsequent structural equation models. Furthermore, structural equation modeling analysis (SEM) was performed in both groups to explore the mediating role of collective self-esteem and ethnic identity between discriminations and

contextual problems considering contextual problems as a unitary construct. Finally, SEM was performed again in both groups separating the contextual problems into three constructs: family, school, and peer. When analyzing goodness-of-fit, by convention, an RMSEA under .08 is acceptable, and under .05 is optimal, while a CFI and TLI higher than .9 are acceptable, and greater than .95 are optimal. SRMR is good below 0.8. According to previous studies, the CMIN ($\chi 2/gl$) is sensitive to sample sizes that go over 200 subjects/participants, although scores under 5 are considered acceptable even with classic criteria [46, 47]. Robust maximum likelihood (MLR) it was the estimator used in SEM, since it is robust even with not-normal distribution.

In this study, data were analyzed using the SPSS version 21.0 and the Mplus version 7.4. G*Power 3.1.9.7 was used to calculated size effect in MANOVA.Cohen [48] suggest the reference values for F^2: 0.02 small effect, 0.15 medium effect, and 0.35 large effect.

Little's MCAR test was used in both groups to analyzed missing data. In both groups, primary and secondary, it was identified a missing not at random data (MNAR). The items from everyday discrimination scale concentrate missing values with a range to 11.5 to 17.7% per item in primary, and 10.1 to15.4% in secondary. It was not found patterns with variables outside the everyday discrimination scale. Multiple imputation (MI) it was used to treatment of missing values; seven imputed data sets were generated in each group sample. In MI the goodness of fit is calculated in each imputed group, and the final result of each index is the average of all groups. This procedure does not allow for confidence intervals, but it does give the standard deviation.

Out-layers were identified in both groups, considering a z-score equal or greater than ± 3. In primary school, 89 cases (6,4%) were found, and 138 cases (6%) in secondary school.

These were not removed from the sample, but the difference between including them or not in the analyses is reported in the result section.

Results

Descriptive statistics and correlations

The asymmetry and kurtosis of both groups are presented in Table 2. It can be seen that the variable with the greatest asymmetry is peer problems. According to Ryu [49] it is acceptable up to 2 for the asymmetry and 7 for the kurtosis to consider using without problems the analysis that assumed the normal distribution.

Table 2. Asymmetry and Kurtosis

	Primary			Secondary	
Variable	Asymmetry	Kurtosis		Asymmetry	Kurtosis
ACOLECT	-.898	1.085		-1.003	.593
DISCRIM	1.286	.822		1.175	1.099
ID_ETNIC	-.045	-.918		.367	-.729
CONTEXT	1.522	2.568		1.164	1.663
P_FAMIL	1.661	3.087		1.092	.977
P_ESCUE	1.339	1.703		1.076	1.046
P_COMPA	2.061	5.049		2.376	7.443

Legend: ACOLECT = collective self-esteem. DISCRIM = perceived discrimination. ID_ETNIC = ethnic identity. CONTEXT = contextual problems. P_FAMIL = family problems. P_ESCUE = school problems. P_COMPA = peer problems.

Furthermore, Table 3 details the values of the means, standard deviations, and correlations of all variables in the study in both groups. It is observed that perceived discrimination, ethnic identity, and collective self-esteem correlate both with the separate

indices of contextual problems and also with contextual problems' general index. The exceptions in both groups are the non-significant correlations between ethnic identity and peer problems, and between ethnic identity and perceived discrimination, while its correlation with the overall index of contextual problems is the lowest of those that are significant.

Table 3. Pearson's correlations. means. and standard deviations of study variables

Primary		ACOLECT	DISCRIM	ID_ETNIC	CONTEXT	P_FAMIL	P_ESCUE	P_COMPA	*M*	*SD*
	ACOLECT	1							32.3	6.2
	DISCRIM	.243**	1						18.5	11.1
	ID_ETNIC	.092**	0.038	1					16.9	6.8
	CONTEXT	-.157**	-.426**	-.070**	1				52.6	10.2
	P_FAMIL	-.128**	-.347**	-.057*	.859**	1			1.6	0.6
	P_ESCUE	-.152**	-.283**	-.087**	.739**	.469**	1		1.7	0.7
	P_COMPA	-.100**	-.378**	-0.031	.754**	.444**	.382**	1	1.5	0.6
Secondary										
	ACOLECT	1							33.9	6
	DISCRIM	-.199**	1						18.8	9.2
	ID_ETNIC	.156**	0.007	1					14.3	6.4
	CONTEXT	-.149**	.446**	-.086**	1				51.4	8.6
	P_FAMIL	-.155**	.312**	-.066**	.768**	1			1.7	0.7
	P_ESCUE	-.113**	.314**	-.156**	.729**	.373**	1		1.8	0.7
	P_COMPA	-.077**	.397**	0.007	.760**	.315**	.387**	1	1.3	0.5

Legend: ACOLECT = collective self-esteem. DISCRIM = perceived discrimination. ID_ETNIC = ethnic identity. CONTEXT = contextual problems. P_FAMIL = family problems. P_ESCUE = school problems. P_COMPA = peer problems.

* $p < 0.05$ (bilateral). ** $p < 0.01$ (bilateral).

Multivariate analysis of variance

A multivariate analysis of variance (MANOVA) was conducted considering gender and age as independent variables in both groups. The dependent variables were collective self-esteem. discrimination. ethnic identity. contextual problems. problems with the family. problems with school and problems with peer. Tables 4 and 5 show the significance of the main effects, and interaction, and the marginal means, respectively, for both groups. The box test is significant in both groups; therefore, the Pillai trace was considered in the global models.

In the primary group, the Pillai trace value was significant for the main effects. being found for sex of F(7.1394) =7.685. p< .001. f^2=.07. and for age of F(14.2790) =3.403. p< .001. f^2=.02. No significant effect was found on the interaction between gender and age being F(42.13710) =1.516. p=.212. f^2=.003. Boys presented greater perceived discrimination, contextual problems in general, as well as problems with school and peer. In ethnic identity the age is significant. being found in the DMS post-hoc tests that the 11-year-old group is different from the other two.

In secondary, the Pillai Trace Value was significant for the main effects. being found a value for gender of F(7.2280) =6.610. p< .001. f^2=.044. and for age of F(42.13710) =5.529. p< .001. f^2=.01. A significant effect was also found for the interaction between gender and age. F(42.13710) =1.516. p< .05. f^2=.001.

The gender differences are in collective self-esteem, perceived discrimination, ethnic identity, general contextual problems, school problems, and peer problems, with ethnic identity being the only one greater in girls, and the rest are all greater in boys. With respect to age, significant differences were found in collective self-esteem. Post-hoc Gabriel tests

found that the 12-year-old group differed significantly in collective self-esteem from the 14. 15. 16 and 17-year-old groups, *p*<.000. The 13-year old group differs from the 15-year old group, *p*<.05, and the 17-year old group, *p*<.000, decreasing in all of them as age increases. An interaction effect between gender and age was obtained. *p*<.05, finding that at 16 years of age, the highest value of collective self-esteem is produced in boys and the lowest in girls.

Table 4. Resume of significant effects

	**Primary**			**Secondary**		
Variable	Gender	Age	Gender * Age	Gender	Age	Gender * Age
ACOLECT	.062	.325	.910	.020*	.000**	.015*
EDST	.000**	.661	.398	.003**	.209	.893
ID_ETNIC	.239	.012**	.058	.028*	.753	.310
PD_CTX	.000**	.409	.104	.006**	.350	.166
PD_FAM	.058	.526	.100	.067	.287	.494
PD_ESC	.000**	.113	.018**	.000**	.492	.935
PD_COM	.012**	.267	.785	.000**	.569	.080

Legend: ACOLECT = collective self-esteem. DISCRIM = perceived discrimination. ID_ETNIC = ethnic identity. CONTEXT = contextual problems. P_FAMIL = family problems. P_ESCUE = school problems. P_COMPA = peer problems.

* p < 0.05 (bilateral). ** p < 0.01 (bilateral).

Note: interaction effect in primary it was not explained since the global model was not significant.

Table 5. Marginal Means of MANOVA

	Primary		Secondary	
	Gender	Age	Gender	Age

Variable	Boys	Girls	9	10	11		Boys	Girls	12	13	14	15	16	17	18
ACOLECT	31.9	32.8	32.5	32.0	32.8		34.3	33.5	35.7	34.9	33.6	33.1	33.5	32.2	34.3
DISCRIM	20.8	16.6	17.8	19.2	18.4		19.7	17.8	17.8	18.7	19.1	19.6	19.2	18.7	16.9
ID_ETNIC	16.6	17.2	17.3	17.5	15.8		14.0	15.0	15.1	14.4	14.4	14.3	14.3	14.2	14.9
CONTEXT	53.0	50.2	50.7	51.3	52.3		51.5	50.1	50.9	51.3	50.7	51.0	51.0	50.6	48.0
P_FAMIL	1.5	1.5	1.5	1.5	1.5		1.7	1.7	1.7	1.7	1.7	1.7	1.7	1.7	1.5
P_ESCUE	1.8	1.5	1.6	1.6	1.7		1.9	1.7	1.7	1.8	1.8	1.8	1.8	1.8	1.7
P_COMPA	1.5	1.4	1.3	1.4	1.4		1.4	1.3	1.4	1.3	1.3	1.3	1.4	1.3	1.2

Legend: ACOLECT = collective self-esteem. DISCRIM = perceived discrimination. ID_ETNIC = ethnic identity. CONTEXT = contextual problems. P_FAMIL = family problems. P_ESCUE = school problems. P_COMPA = peer problems.

Analysis of the unitary model of contextual problems

Unitary SEM in the primary group

Fig 1 shows the unitary model of contextual problems in the primary group. It can be seen that ethnic identity does not show a mediation effect since it is not affected by perceived discrimination. At the same time, collective self-esteem does appear as a mediator between perceived discrimination and contextual problems, as well as between ethnic identity and contextual problems. Ethnic identity does have a direct effect on collective self-esteem (r = .160; p<.001); Perceived discrimination does have a direct effect on contextual problems (r = .377; p<.001); and a negative direct effect on collective self-esteem (r = -.203; p<.001); there is a significant indirect effect of ethnic identity on contextual problems (r = -0.020; p<.005); and a significant indirect effect of perceived discrimination on contextual problems (r = 0.025; p<.005). Age presented a direct effect on collective self-esteem (r = .057; p<.001); Gender presented a direct effect on contextual problems (r = .067; p<.001), finding that being a boy predicts greater problems. Finally, collective self-esteem presents a negative direct

effect on contextual problems (r = -.124; p<.001). Regarding this analysis. goodness-of-fit indicators are optimal. as demonstrated herein: $\chi 2/gl$ = 2.13. CFI = .981. TLI = .977. RMSEA = 0.028 (SD = .001), AIC = 62393.516, BIC = 62634.355, SRMR = 0.035.

Figure 1. Primary SEM considering contextual problems as a unitary construct with their standardized values. Legend: Identic = ethnic identity. Discri = perceived discrimination. Aucole = collective self-esteem. Pd_pctx = Contextual Problems.

Unitary SEM in the secondary group

Fig 2 shows the unitary model of contextual problems in the secondary group. It can be seen that ethnic identity does not show a mediation effect since it is not affected by perceived discrimination, but it shows a negative direct effect on contextual problems(r = -.096; p<.05); there is also a significant negative indirect effect of ethnic identity on contextual problems (r = -0.011; p<.05). Further, ethnic identity presented a direct effect on collective self-esteem (r = .196; p<.001). At the same time, collective self-esteem does appear as a mediator between perceived discrimination and contextual problems, as well as between ethnic identity and contextual problems. Furthermore, perceived discrimination does have a direct effect on contextual problems (r = .453; p<.001); and a significant indirect effect on the same variable (r = 0.010; p<.01). Perceived discrimination also presented a negative direct effect on collective self-esteem (r = -.167; p<.001). Collective self-esteem presented a negative direct effect on contextual problems (r = -.058; p<.01); Age presented a negative direct effect on collective self-esteem (r = -.179; p<.001), and a negative direct effect on

contextual problems (*r* = -.071; *p*<.01); Gender presented a direct effect on collective self-esteem (*r* = .064; *p*<.001), finding that being a boy predicts better collective self-esteem. Regarding this analysis, goodness-of-fit indicators are optimal, as demonstrated herein: χ2/gl = 4.66, CFI = .973, TLI = .967, RMSEA = 0.040 (*SD* = .001), AIC = 94034.088, BIC = 94304.326, SRMR = 0.031.

Figure 2. Secondary SEM considering contextual problems as a unitary construct with their standardized values. Legend: Identic = ethnic identity. Discri = perceived discrimination. Aucole = collective self-esteem. Pd_pctx = Contextual Problems.

Divided model analysis of contextual problems

Divided SEM in the primary group

Fig 3 shows the standardized values of the contextual problem model separated by family, school, and peer. In this analysis, ethnic identity is observed to have a negative direct effect on school problems (*r* = -.064; *p*<.05), and it also has a negative indirect effect, mediated by collective self-esteem, on family problems (*r* = -.016; *p*<.01), and on school problems (*r* = -.019; *p*<.01), and peer problems (*r* = -.013; *p*<.01). Ethnic identity does have a direct effect on collective self-esteem (*r* = .159; *p*<.001). Further, perceived discrimination has a direct effect on the three types of contextual problems, being greater in peer problems (*r* = .328; *p*<.001). followed by family problems (*r* = .292; *p*<.001), and by school problems (*r* = .256; *p*<.001). Therefore, perceived discrimination also presented a negative direct effect on collective self-esteem (*r* = -.198; *p*<.001). In turn, there is an indirect effect of perceived

discrimination on family problems (r = .020; p<.01), and on school problems (r = .024; p<.01), and on peer problems (r = .016; p<.05). Collective self-esteem is observed to have a negative direct effect in all specific contextual problems, being greater in school problems (r = -.120; p<.001), followed by family problems (r = -.102; p<.01), and by peer problems (r = -.083; p<.01). Age shows a direct effect on school problems (r = .090; p<.001), and collective self-esteem (r = .057; p<.05). Gender presented a direct effect in school problems (r = .120; p<.001), finding that being a boy predicts greater problems. Regarding this analysis, goodness-of-fit indicators are optimal, as demonstrated herein: $\chi 2$/gl = 2.17, CFI = .980, TLI = .972, RMSEA = .029 (*SD* = .001), AIC = 59326.853, BIC = 59667.169, SRMR = 0.032.

Figure 3. Primary SEM considering contextual problems as separate constructs. with their standardized values. Legend: Identic = ethnic identity. Discri = perceived discrimination. Aucole = collective self-esteem. Pd_famil = family problems. Pd_escue = school problems. Pd_compa = peer problems.

Divided SEM in the secondary group

Fig 4 shows the standardized values of the contextual problem model separated by family, school, and peer. In this analysis, ethnic identity is observed to have a negative direct effect on family problems (r = -.070; p<.01), and school problems (r = -.161; p<.001), and it also has a negative indirect effect, mediated by collective self-esteem, on family problems (r = -.013; p<.01). Ethnic identity does have a direct effect on collective self-esteem (r = .196; p<.001). Further, perceived discrimination has a direct effect on the three types of contextual

problems, being greater in peer problems (r = .389; p<.001). followed by school problems (r = .347; p<.001), and by family problems (r = .301; p<.001). Therefore, perceived discrimination also presented a negative direct effect on collective self-esteem (r = -.167; p<.001). In turn, there is an indirect effect of perceived discrimination family problems (r = .011; p<.01). Collective self-esteem is observed to have a negative direct effect on family problems (r = -.069; p<.01). Age shows a negative direct effect on peer problems (r = -.059; p<.001). and collective self-esteem (r = -.179; p<.001). Gender presented a direct effect in all specific contextual problems, being greater in peer problems (r = .102; p<.001), followed by a negative effect on family problems (r = -.095; p<.001), and by school problems (r = .066; p<.001). Finding that being a boy predicts greater peer and school problems, and being a girl predicts greater family problems. Regarding this analysis, goodness-of-fit indicators are optimal. as demonstrated herein: $\chi 2$/gl = 4.85. CFI = .968, TLI = .958, RMSEA = .041 (*SD* = .001), AIC = 89266.287, BIC = 89628.521, SRMR = 0.030.

Figure 4. Secondary SEM considering contextual problems as separate constructs. with their standardized values. Legend: Identic = ethnic identity. Discri = perceived discrimination. Aucole = collective self-esteem. Pd_famil = family problems. Pd_escue = school problems. Pd_compa = peer problems.

Goodness-of-fit for all models are optimal since all parameters are beyond the acceptable ranges, and some even beyond the optimal ranges. Finally, collective self-esteem was regarded with a role of partial mediation for both ethnic identity and perceived discrimination in all models.

Effect to removes out-layers

The results of removing the out-layers before multiple imputation should be considered as reference. In both primary and secondary groups, the unitary model, all the effects in relationships are maintained, and the maximum reduction of the effects reaches .03.

In the specific primary model, all relationships are maintained, except for the effect of ethnic identity on school problems, which is no longer significant. A gender relationship appears on peer problems (.06). The maximum effect variation in all relationships is around .03, except for the effect of perceived discrimination on school problems, which decreases by .07. In the specific model for secondary schools, all relationships are maintained, and the maximum variation in the effects on relationships is .04. In all these SEM models, optimum goodness fit indicators are maintained.

Discussions

The aim of this study was divided into two sections: first. to establish the effects of the relation between perceived discrimination and contextual problems in inter-ethnic students aged 9 to 19 living in the northern region of Chile; and second, to identify the role that ethnic identity and collective self-esteem play within this relation. The three hypotheses that derived from this objective were: 1) The greater the perceived discrimination, the greater the contextual problems both in general and specifically. 2) Ethnic identity has a mediating role between perceived discrimination and contextual problems, both in general and specifically. 3) Collective self-esteem has a mediating role between perceived discrimination and contextual problems, both in general and specifically.

Relation between perceived discrimination and contextual problems

It was found evidence that fully supports the first hypothesis in both groups. The results indicated that perceived discrimination did have an effect on contextual problems, whether considered as a whole or separately, so it can be inferred that exacerbated discriminatory behaviors towards students may increase their family, school, and peer problems. Further, when considered separately, it can be noted that peer problems was the most affected area by perceived discrimination. In opposition to this finding, early studies on the ecological model gave more importance to students' school relations with their teachers/school than with their peer [21. 22]. However, since our findings demonstrate that peer problems is the most affected area when students experience discriminatory behaviors, it seems necessary to give this type of relation at least an equally important role to that of teachers/school when theorizing the importance of school relations for students. Further, our results coincide with recent research on children and adolescent's development, which consider peer relations equally as important as family and teachers/school relations [24. 25].

In addition, there are newer propositions regarding the ecological model which stated that humans develop through progressively more complex reciprocal interactions, which involve an individual (in this case. the student) and the objects, symbols and individuals in his/her external environment (in this case. family. school and peer), and that, for these interactions to effectively impact the individual's development. these interactions must be regular and placed over extended periods of time; this new concept was called proximal process [50]. Thus, based on the concepts of proximal processes, if we consider that peer spend much more time with the student than their parents or teachers/school, it is quite logical to assume that their role is much more relevant than what this same theory originally stated

and, therefore, it seems relevant to pay attention to the effects of perceived discrimination on peer problems when analyzing children and adolescent development.

Moreover, peer relations and problems are generally studied within the perspective of bullying [51]. However, this is a limitation in this study because there are other conflicts that do not necessarily involve students being bullied and still involve discriminatory behaviors. Thus, further investigations on these relations are needed since they are relevant for children's and adolescents' development and may even be central in that regard.

Now. when taking into account the direct effect of perceived discrimination on family problems, we found that it is consistent with the results obtained by previous research [11. 19], although they used different instruments to measure family problems. These findings are also in line with the results obtained by a study conducted with an adult Latin American population [18].

As for school problems, a review study noted that previous studies have usually investigated the student-teacher relationship in the context of teacher discriminatory behavior or in aspects not directly related to conflict, such as school climate investigations; and that previous studies have usually investigated the direct student-school relation in the contexts of size, culture, and safety of the school for the students [23]. Thus, since this study's findings indicate that school problems are also affected by discrimination. it seems necessary for further studies to investigate school problems, as it may further improve the understanding of children's and adolescents' development.

It should be noted that in unitary models, the direct effect of perceived discrimination on contextual problems increases by .07 in the secondary group. Likewise, in divided models the effect of discrimination on school and peer problems increases by .06 or more, but only .01 in family problems. It is possible to hypothesize that secondary school students have

experienced a cumulative effect of perceived discrimination, and this increases the direct effect of discrimination on contextual problems. Future research may address this possibility.

Ethnic identity and collective self-esteem role.

The second hypothesis was not supported by evidence since ethnic identity was not affected by perceived discrimination in any group. Furthermore, in the unitary models of both groups, ethnic identity neither had no significant effect on contextual problems. In the divided models of both groups, it was not related to all specific contextual problems, but to some. The present results rule out the mediation of ethnic identity in the relation between perceived discrimination and contextual problems, although it may have presented a direct effect on some contextual problems in the divided model's analysis. In both groups, ethnic identity has a direct effect on school problems, but only in the secondary group also affect to family problems. These results are supported by research which stated that perceived discrimination is not significantly related to the ethnic identity of adolescents, although they may have used different instruments to measure discrimination [52, 53].

However, one of these previous studies has brought up another variable into this question: maybe ethnic identity has not matured enough in adolescents, so it would not establish such a relation with discrimination due to it still being in development [52]. In partial corroboration to that notion, another study performed on Latin American adults was able to effectively find a relation between both variables (ethnic identity and perceived discrimination) [54]. On the other hand, another study performed on Asian American adults was not able to effectively find a relation between both variables [55].

Notwithstanding, some of these results partially contradict what a previous study found using the same MEIM-R scale that was used in this study, as a relation between

perceived discrimination and ethnic identity in adolescents was found [56]. In addition, other studies that did not necessarily use this scale have also found solid relations between both variables in adolescents [28. 31. 57]. In short, there are mixed results that do not allow to either discard or affirm this relation, and this has been previously highlighted in a previous meta-analysis study [44].

Furthermore, the direct effect of ethnic identity on family problems found in this research contradicts the findings of previous research, which stated that ethnic identity significantly affected parent-child relations in African Americans, but not in Latin Americans [33].

Evidence was found that partially supports the third hypothesis of the study, which affirms the collective self-esteem mediation relationship between discrimination and contextual problems. In unitary models of both groups, mediation appears, but in divided models, it does not appear completely, because collective self-esteem affects all contextual problems only in the primary school group. In the secondary group, it affects exclusively family problems.

Moreover, the negative effect of discrimination on collective self-esteem is expected and is consistent with previous research conducted with adolescents [26. 28], as well as those conducted with children [58]. However, in another study, a relation between the two variables was not found in children, but a different instrument was used to measure discrimination, and that should be taken into account when comparing both [59].

Additionally, the relation between ethnic identity and collective self-esteem coincides with the results of a previous study which found a positive relation between the two [28] and another study that found that ethnic identity predicts collective self-esteem [37]. Furthermore, it is logical to situate both variables within the macrosystem of

Bronfenbrenner's theory since they form part of the culture in which the individual is immersed.

Further, this research had the opportunity of including latent variables, which implies that we did consider the statistical measurement error through an adequate treatment of missing values – by multiple imputation (MI) in the present study - taking into account the recommendations of previous studies [60]. This same study emphasized that significant statistical effects should not be discarded, even if they were modest, but it is quite clear that the role of mediation of collective self-esteem does not seem sufficient to focus strategized interventions on it. On the other hand, perceived discrimination and ethnic identity seemed as more reasonable focal points when creating strategized intervention efforts, and since both ethnic identity and collective self-esteem are linked to the individual's identity, it seems reasonable to include the measurement of identity when strategizing these interventions.

In terms of strengths, the models of structural equations proposed in this study had appropriate goodness-of-fit indicators, and specifics models provided relevant findings for this study's aim. Another strength was the wide age range of participants, and this is a relevant aspect because this type of study usually investigates either adolescents or children, not both.

Nonetheless, this study had several limitations. In terms of data gathering, convenience sampling was performed, so it reduced the possibilities of result generalization, and further studies should try to select more specific sample groups to provide more accurate notions regarding the topic of study. Further, only one region of the country was considered and incorporated, so further studies that encompass a more geographically varied sample would be ideal. Moreover, although the SEM models were guided and performed based on previous theories, they did not offer experimental evidence that allows for the establishment of causality among the variables. Future studies should try to utilize statistical models that

may allow for the establishment of causality among the variables to further improve the range of conclusions of the study. Finally, this study also relies exclusively on student's self-reports, so other studies that offer different types of reports and observations in terms of data collection are required to further investigate perceived discrimination among children and adolescents in Chile. Furthermore, although the SENA instrument has an inconsistency scale, it does not have a social desirability scale.

Finally, regarding future researches, we believe that there are some specific changes regarding the methodological approach that could further contribute to the understanding of the studied phenomenon, and are described herein: including other contextual aspects and problems, such as the children's and adolescents' neighborhood; the inclusion of other variables, such as personal self-esteem in the statistical model; the inclusion of others' perspectives regarding the discriminatory process, such as the parents' and teachers' (an action that would be consistent with the ecological model that was explored in this study); and changing the study's perspective to a longitudinal one.

Conclusions

In conclusion, this study showed that perceived discrimination affects contextual problems - whether considered as a whole or separately - at the family, school and peer levels; ethnic identity has a direct effect on school problems in primary and secondary students, and in this last group also in family problems; and collective self-esteem has a mediating role for both ethnic identity and perceived discrimination over contextual problems.

Acknowledgements

This research has been funded by ANID PIA CIE160007.

References

1. Instituto Nacional de Estadísticas. Departamento de Demografía y Censos (2018) Características de la inmigración internacional en Chile. Censo 2017. Available from: http://www.censo2017.cl/descargas/inmigracion/181123-documento-migracion.pdf
2. Ministerio de Educación. Centro de Estudios (2018). Mapa del estudiantado extranjero en el sistema escolar chileno (2015-2017). Documento de trabajo N° 12. Santiago. Chile. Available from: https://www.mineduc.cl/wp-content/uploads/sites/19/2018/05/MAPA_ESTUDIANTES_EXTRANJEROS_SISTEMA_ESCOLAR_CHILENO_2015_2017.pdf
3. de Aquino RIC. Wiesner MLR. García AAE. Moreno AS. Educación inclusiva y niñez migrante: barreras a la educación de niñez de origen centroamericano en escuelas de Tapachula. Chiapas (México). Revista de Investigación en Educación. 2016;14(1): 85-104.
4. United Nations General Assembly. Convention on the Rights of the Child. [cited 11 June 2019] United Nations. Treaty Series. 1989;1577: 3. Available from: https://www.refworld.org/docid/3ae6b38f0.html.
5. Guizardi M. Las fronteras del transnacionalismo. 1st ed. Santiago de Chile: Ocho libros; 2015.
6. Hopenhayn M. Bello A. Discriminación étnico-racial y xenofobia en América Latina y el Caribe. Santiago de Chile: CEPAL; 2001.

7. Neblett EW. Rivas- Drake D. Umaña- Taylor AJ. The promise of racial and ethnic protective factors in promoting ethnic minority youth development. Child Devel Perspec. 2012;6(3): 295-303. doi: 10.1111/j.1750-8606.2012.00239.x.
8. Tropp. L.R. O'Brien TC. Gutierrez RG. Valdenegro D. Migacheva K. de Tezanos-Pinto P. et al. How school norms. peer norms. and discrimination predict interethnic experiences among ethnic minority and majority youth. Child Devel. 2016;87(5): 1436-1451. doi: 10.1111/cdev.12608.
9. Busse D. Yim IS. Campos B. Social context matters: Ethnicity. discrimination and stress reactivity. Psychoneuroendocrinology. 2017;83: 187–193. doi: 10.1016/j.psyneuen.2017.05.025.
10. Pachter LM. Caldwell CH. Jackson JS. Bernstein BA. Discrimination and mental health in a representative sample of African-American and Afro-Caribbean youth. J Racial Ethnic Health Disp. 2017;5(4): 831-837. doi: 10.1007/s40615-017-0428-z.
11. Ríos-Salas V. Larson A. Perceived discrimination. socioeconomic status. and mental health among Latino adolescents in U.S. immigrant families. Children and Youth Serv Rev. 2015;56: 116-125. doi: 10.1016/j.childyouth.2015.07.011.
12. Walsh SD. Kolobov T. Simanovskaya O. What is it about perceived discrimination that can lead immigrant adolescents to alcohol use and delinquency? The mediating role of feelings of alienation. Subst Use Misuse. 2019;54(1): 65-77. doi: 10.1080/10826084.2018.1495738.
13. Becerra S. Merino M. Mellor D. Ethnic discrimination against Mapuche students in urban high schools in the Araucanía region. Chile. Inter Educ Stud. 2015;8(10). doi: 10.5539/ies.v8n10p96.

14. Lahoz S. Forns M. Discriminación percibida. afrontamiento y salud mental en migrantes peruanos en Santiago de Chile. Psicoperspectivas. 2015;15(1): 157-168. doi: 10.5027/PSICOPERSPECTIVAS-VOL15-ISSUE1-.

15. Cheng HL. Lin SP. Cha CH. Perceived discrimination. intergenerational family conflicts. and depressive symptoms in foreign-born and U.S.-born Asian American emerging adults. Asian American J Psychol. 2015;6(2): 107-116. doi: 10.1037/a0038710.

16. Juang L.P. Alvarez AA. Discrimination and adjustment among Chinese American adolescents: Family conflict and family cohesion as vulnerability and protective factors. Am J Public Health. 2010;100(12): 2403-2409. doi: 10.2105/AJPH.2009.185959.

17. Juang L.P. Alvarez AA. Family. school. and neighborhood: Links to Chinese American adolescent perceptions of racial/ethnic discrimination. Asian Am J Psychol. 2011;2(1): 1-12. doi: 10.1037/a0023107.

18. Molina KM. Little T.V. Rosal MC. Everyday discrimination. family context. and psychological distress among Latino adults in the United States. J Comm Psychol. 2016;44(2): 145-165. doi: 10.1002/jcop.21747.

19. Ponting C. Steve SS. Escovar E.L. Rappa AM. Camacho A. Calderon I. et al. Family factors mediate discrimination related stress and externalizing symptoms in rural Latino adolescents. J Adolesc. 2018;69: 11-21. doi: 10.1016/j.adolescence.2018.08.007.

20. Wong CA. Eccles JS. Sameroff A. The influence of ethnic discrimination and ethnic identification on African American adolescents' school and socioemotional adjustment. J Pers. 2003;71(6): 1197-1232. doi: 10.1111/1467-6494.7106012

21. Bronfenbrenner U. The ecology of human development. Cambridge: Harvard University Press; 1979.
22. Bronfenbrenner U. Discovering what families do. In: Blankenhorn D. Bayme S. Elshtain J.B. Rebuilding the nest: A new commitment to the American family. Milwaukee: Family Service America; 1990. pp. 27-38.
23. Eccles JS. Roeser RW. Schools as developmental contexts during adolescence. J Res Adolesc. 2011;21(1): 225–241. doi: 10.1111/j.1532-7795.2010.00725.x.
24. Preiss DD. Calcagni E. Grau V. Classroom research and child and adolescent development in South America. New Dir Child Adolesc Dev. 2015;2015(147): 85-92. In: Jensen LA. Larson RW. The global context for new directions for child and adolescent development PREFACE. New Dir Child Adolesc Dev. 2015;147: 1-2. doi: 10.1002/cad.20090.
25. Hughes DL. Watford JA. Del Toro J. A transactional/ecological perspective on ethnic–racial identity. socialization. and discrimination. Adv Child Dev Behav. 2016;51: 1-41. In: Horn S. Ruck M. Liben L. Advances in child development and behavior. Volume 51: Equity and justice in developmental science: Implications for young people. families. and communities. 1st ed. San Diego: Elsevier Academic Press; 2016. Pp. 1-41.
26. Jia X. Liu X. Shi B. Perceived discrimination and subjective well-being in Chinese migrant adolescents: Collective and personal self-esteem as mediators. Front Psychol. 2017;8: 1213. doi: 10.3389/fpsyg.2017.01213.
27. Luhtanen R. Crocker J. A Collective Self-Esteem Scale: Self-evaluation of one's social identity. Pers Social Psychol Bull. 1992;18(3): 302-318.

28. Liu X. Zhao J. Chinese migrant adolescents' perceived discrimination and psychological well-being: The moderating roles of group identity and the type of school. PLOS ONE. 2016;11(1): e0146559. doi: 10.1371/journal.pone.0146559.
29. Tajfel H. Grupos humanos y categorías sociales. Barcelona: Herder; 1984.
30. Cavanaugh AM. Stein GL. Supple AJ. Gonzalez LM. Kiang L. Protective and promotive effects of Latino early adolescents' cultural assets against multiple types of discrimination. J Res Adolesc. 2018;28(2): 310-326. doi: 10.1111/jora.12331.
31. Marcelo AK. Yates TM. Young children's ethnic–racial identity moderates the impact of early discrimination experiences on child behavior problems. Cultur Divers Ethnic Minor Psychol. 2019;25(2): 253-265. doi: 10.1037/cdp0000220.
32. Romero AJ. Edwards LM. Fryberg SA. Orduña M. Resilience to discrimination stress across ethnic identity stages of development. J App Soc Psychol. 2013;44(1): 1-11. doi: 10.1111/jasp.12192.
33. Hurwich- Reiss E. Rienks SL. Bianco H. Wadsworth ME. Markman HJ. Exploring the role of ethnic identity in family functioning among low- income parents. J Comm Psychol. 2015;43(5): 545–559. doi: 10.1002/jcop.21701.
34. Torres SA. Santiago CD. Culture and educational stress and internalizing symptoms among Latino adolescents: The role of ethnic identity. J Edu Psychol Consult. 2017;27(3): 344–366. doi: 10.1080/10474412.2017.1301819.
35. Jiménez-Bustos RA. Arenas RXPL. Rivera GEB. Identidad étnica y conductas sociales en adolescentes indígenas Mapuche sancionados por la Ley de Responsabilidad Penal Adolescente en regiones del sur de Chile. Univ Psychol. 2017;16(1): 1-22. doi: 10.11144/Javeriana.upsy16-1.iecs.

36. González R. Lickel B. Gupta M. Tropp LR. Luengo Kanacri BP. Mora E. et al. Ethnic identity development and acculturation preferences among minority and majority youth: Norms and Contact. Child Dev. 2017;88(3): 743-760. doi: 10.1111/cdev.12788.

37. Jetten J. Branscombe N.R. Haslam SA. Haslam C. Cruwys T. Jones JM. et al. Having a lot of a good thing: Multiple important group memberships as a source of self-esteem. PLOS ONE 2015;10(5): e0131035. doi: 10.1371/journal.pone.0124609.

38. Williams DR. Yu Y. Jackson JS. Anderson NB. Racial differences in physical and mental health: Socioeconomic status. stress. and discrimination. J Health Psychol. 1997;2(3): 335-351. doi: 10.1177/135910539700200305.

39. Krieger N. Smith K. Naishadham D. Hartman C. Barbeau EM. Experiences of discrimination: validity and reliability of a self-report measure for population health research on racism and health. Soc Sci Med. 2005;61(7): 1576-1596.

40. Espinosa A. Acosta Y. Valencia J. Vera A. Soares da Silva A. Romero JC. et al. Calidez. competencia. moralidad y nacionalismo ideal como dimensiones autoestereotípicas del autoconcepto nacional en seis países de Latinoamérica. Avances en Psicología Latinoamericana. 2006;34(2): 395-413.

41. Fernández-Pinto I. Santamaría P. Sánchez-Sánchez F. Carrasco MA. del Barrio V. Sistema de evaluación de niños y adolescentes. Manual de aplicación. corrección e interpretación. Madrid: TEA Ediciones; 2015.

42. Sánchez-Sánchez F. Fernández-Pinto I. Santamaría P. Carrasco MA. del Barrio V. SENA. Sistema de Evaluación de Niños y Adolescentes: proceso de desarrollo y evidencias de fiabilidad y validez. Revista de Psicología Clínica con Niños y Adolescentes. 2016;3(2): 23-34.

43. Phinney JS. Ong AD. Conceptualization and measurement of ethnic identity: Current status and future directions. J Couns Psychol. 2007;54(3): 271-281. doi: 10.1037/0022-0167.54.3.271.

44. Rivas-Drake D. Syed M. Umaña-Taylor A. Markstrom C. French S. Schwartz SJ. et al. Feeling good. happy. and proud: A meta-analysis of positive ethnic-racial affect and adjustment. Child Dev. 2014;85(1): 77–102. doi:10.1111/cdev.12175.

45. Lara, L. & Martínez-Molina, A. (2016). Validación de la Escala de Identidad étnica Multigrupo-Revisada en adolescentes inmigrantes y autóctonos residentes en España. *Revista Latinoamericana de Ciencias Sociales, Niñez y Juventud*, *14* (1), pp. 591-601. http://dx.doi.org/10.11600/1692715x.14140110515

46. Brown TA. Methodology in the social sciences. Confirmatory factor analysis for applied research. 2nd ed. New York: The Guilford Press; 2015.

47. McDonald RP. Moon-Ho RH. Principles and practice in reporting structural equation analyses. Psychol Meth. 2002;7(1): 64-82. doi: 10.1037/1082-989X.7.1.64.

48. Cohen J. Statistical power analysis for the behavioral sciences. Abingdon. England: Routledge. 1988.

49. Ryu E. Effects of skewness and kurtosis on normal-theory based maximum likelihood test statistic in multilevel structural equation modeling. Behavior Research Methods, 2011;43(4), 1066-1074. doi: 10.3758/s13428-011-0115-7

50. Bronfenbrenner U. Morris PA. The bioecological model of human development. In: Lerner RM. Damon W. Handbook of child psychology: Theoretical models of human development. Hoboken: John Wiley & Sons Inc; 2006. pp. 793-828.

51. Lorenzo-Blanco EI. Unger JB. Oshri A. Baezconde-Garbanati L. Soto D. Profiles of bullying victimization. discrimination. social support. and school safety: Links with

Latino/a youth acculturation. gender. depressive symptoms. and cigarette use. Am J Orthopsychiatry. 2016;86(1): 37-48. doi: 10.1037/ort0000113.

52. Myrick SE. Martorell GA. Sticks and stones may break my bones: Protective factors for the effects of perceived discrimination on social competence in adolescence. Pers Rel. 2011;18(3): 487-501. doi: 10.1111/j.1475-6811.2010.01320.x.
53. Tummala-Narra P. Claudius M. Perceived discrimination and depressive symptoms among immigrant-origin adolescents. Cultur Divers Ethnic Minor Psychol. 2013;19(3): 257-269. doi: 10.1037/a0032960.
54. Molina KM. Estrella ML. Durazo-Arvizu R. Malcarne VL. Llabre MM. Isasi CR. et al. Perceived discrimination and physical health-related quality of life: The Hispanic Community Health Study/Study of Latinos (HCHS/SOL) Sociocultural Ancillary Study. Soc Sci Med. 2019;222: 91-100. doi: 10.1016/j.socscimed.2018.12.038.
55. Yoo H. Lee R. Ethnic identity and approach-type coping as moderators of the racial discrimination/well-being relation in Asian Americans. J Couns Psychol. 2005;52(4): 497-506. doi: 10.1037/0022-0167.52.4.497.
56. Cristini F. Scacchi L. Perkins D.D. Santinello M. Vieno A. The influence of discrimination on immigrant adolescents' depressive symptoms: What buffers its detrimental effects? Psychosoc Interv. 2011;20(3): 243-253. doi: 10.5093/in2011v20n3a2.
57. Romero AJ. Roberts RE. The impact of multiple dimensions of ethnic identity on discrimination and adolescents' self-esteems. J App Soc Psychol. 2003;33(11): 2288–2305. doi: 10.1111/j.1559-1816.2003.tb01885.x.

58. Cassidy C. O'Connor RC. Howe C. Warden D. Perceived discrimination and psychological distress: The role of personal and ethnic self-esteem. J Couns Psychol. 2004;51(3): 329–339. doi: 10.1037/0022-0167.51.3.329.
59. Lam BT. Impact of perceived racial discrimination and collective self-esteem on psychological distress among Vietnamese-American college students: sense of coherence as mediator. Am J Orthopsychiatry. 2007;77(3). 370–376. doi: 10.1037/0002-9432.77.3.370.
60. Wolf EJ. Harrington KM. Clark SL. Miller. M.W. Sample size requirements for structural equation models: An evaluation of power. bias. and solution propriety. Educ Psychol Meas. 2013;76(6): 913-934. doi: 10.1177/0013164413495237.

PONE-D-20-05463
Perceived discrimination and contextual problems among children and adolescents in
Northern Chile
PLOS ONE

Dear Jerome Aníbal,

Thank you for submitting your manuscript to PLOS ONE. After careful consideration,
we feel that it has merit but does not fully meet PLOS ONE’s publication
criteria as it currently stands. Therefore, we invite you to submit a revised
version of the manuscript that addresses the points raised during the review
process.

We would appreciate receiving your revised manuscript by 16/07/2020. When you are
ready to submit your revision, log on to
https://www.editorialmanager.com/pone/ and
select the 'Submissions Needing Revision' folder to locate your manuscript
file.

If you would like to make changes to your financial disclosure, please include your
updated statement in your cover letter.

To enhance the reproducibility of your results,
we recommend that if applicable you deposit your laboratory protocols in
protocols.io, where a protocol can be assigned its own identifier (DOI) such that it
can be cited independently in the future. For instructions see:
http://journals.plos.org/plosone/s/submission-guidelines#loc-laboratory-protocols

Please include the following items when submitting your revised manuscript:A
rebuttal letter that responds to each point raised by the academic editor and
reviewer(s). This letter should be uploaded as separate file and labeled
'Response to Reviewers'.A marked-up copy of your manuscript that highlights
changes made to the original version. This file should be uploaded as separate file
and labeled 'Revised Manuscript with Track Changes'.An unmarked version of
your revised paper without tracked changes. This file should be
uploaded as separate file and labeled 'Manuscript'.
Please note while forming your response, if your article is accepted, you may have
the opportunity to make the peer review history publicly available. The record will

include editor decision letters (with reviews) and your responses to reviewer
comments. If eligible, we will contact you to opt in or out.
We look forward to receiving your revised manuscript.

Kind regards,

Eduardo Fonseca-Pedrero, PhD
Academic Editor
PLOS ONE

Journal Requirements:When submitting your revision, we need you to address these
additional requirements.
1. Please ensure that your manuscript meets PLOS ONE's style requirements,
including those for file naming. The PLOS ONE style templates can be found at
https://journals.plos.org/plosone/s/file?id=wjVg/PLOSOne_formatting_sample_main_body.pdf
and
https://journals.plos.org/plosone/s/file?id=ba62/PLOSOne_formatting_sample_title_authors_affiliations.pdf
2. We note that you have stated that you will provide repository information for
your data at acceptance. Should your manuscript be accepted for publication, we will
hold it until you provide the relevant accession numbers or DOIs necessary to access
your data. If you wish to make changes to your Data Availability statement, please
describe these changes in your cover letter and we will update your Data
Availability statement to reflect the information you provide.
3. Please use periods "." instead of commas"," for decimal points.
4. Please include additional information regarding the survey or questionnaire used
in the study and ensure that you have provided sufficient details that others could
replicate the analyses. For instance, if you developed a questionnaire as part of
this study and it is not under a copyright more
restrictive than CC-BY, please include a copy, in both the original language and
English, as Supporting Information. Please clarify whether you used a Spanish
version of the MEIM-R and if so how it was validated.

Additional Editor Comments (if provided):

The work entitled "Perceived discrimination and contextual problems among
children and adolescents in Northern Chile” is a good research paper. However,
I have a few comments to make that should be addressed before I recommend this
manuscript for publication to PLOS ONE:

Dear Editor and reviewers:

We are grateful of all comments. We decide to divide the sample in two groups, primary and secondary, since we used different SENA age version in each one. Even if both SENA version share more than 70% items, slight differences in the rest can affect the analysis, and the secondary version has one item more.

1.- Please, add empirical information in the abstract.

It was adding now

2.- Please, add an operative definition of the main constructs used.

There is not a cut-off point for to establish enough collective self-esteem or ethnic identity. Then it was not specified. SENA has cut-off for four risk zones, but it was not used because a continuous variable can show clearer the relationship.

3.- In English, a decimals point is used, not comma.

It was changed

4.- Please, add the main hypothesis at the end of the introduction.

We added three hypotheses

5.- Add more information about sample and sampling procedure as well as others variables like: % by age, socio-economic level, IQ, previous mental disorders, etc. Add all the sample information in the method section, not as a results.

It was added all information and change the position in the document. We don't ask socioeconomic level to child's because the primary group probably don't know

6.- Do you have any information about non-response? Describe inclusion/exclusion criteria if part of the data was excluded from the analysis.

We added criteria

7.- Were outliers removed from the data? Which method did you use to deal with
missing data in the analyses? What variables are related to missing data?

We identified out-layers in both groups, but we dint remove. We explain the differences between include or not at the end of result section. The effects of removing the out-layers are considered. The vast majority of the relationships are maintained and vary in a range of 3-5%. Only one relationship ceases to be significant, ethnic identity on school problems in primary group. In sum, this means that relationships are not generated by the presence of out-layers. In any case, by removing them and recalculating the Z-scores, new out-layers appear. Since the MLR estimator that is resistant to non-normality has been chosen, it is decided to keep all the cases in the sample.

8.- Add information about the psychometric properties of the measures used in this study. In addition, add Omega values, not Cronbach. Cronbach alpha has a lot of limitations from a psychometric point of view.

We added all information about each scale and omega indices for all

9.- Add subheadings in the results section (as function of the goals).
We added subheadings in this section

10.- Add more information about mediation analyses performed.

We added all information

11.- Please, before to SEM analysis, compute a MANOVA or MANCOVA in mean comparisons by gender and age. Add effect sizes in mean comparisons.

We added a MANOVA and effect sizes

12.- Please, add other godness of fit indices, eg.: TLI, BIC, IC RMSEA 90%, etc. Add information about
goodness of fit indices. Test model assumptions (e.g., normality).

We added information about normality, and more fit indices

13.- Add more limitations (e.g., self-reports, no scale to test social desirability).

We added

14.- Check English grammar.

We reviewed it

[Note: HTML markup is below. Please do not edit.]

Reviewers' comments:

Reviewer's Responses to Questions

Comments to the Author

1. Is the manuscript technically sound, and do the data support the conclusions?

The manuscript must describe a technically sound piece of scientific research with
data that supports the conclusions. Experiments must have been conducted rigorously,
with appropriate controls, replication, and sample sizes. The conclusions must be
drawn appropriately based on the data presented.

Reviewer #1: Partly

Reviewer #2: Yes

2. Has the statistical analysis been performed appropriately and rigorously?

Reviewer #1: Yes

Reviewer #2: Yes

3. Have the authors made all data underlying the findings in their manuscript fully available?

The PLOS Data policy requires authors to make all data underlying the findings described in their manuscript fully available without restriction, with rare exception (please refer to the Data Availability Statement in the manuscript PDF file). The data should be provided as part of the manuscript or its supporting information, or deposited to a public repository. For example, in addition to summary statistics, the data points behind means, medians and variance measures should be available. If there are restrictions on publicly sharing data—e.g. participant privacy or use of data from a third party—those must be specified.

Reviewer #1: Yes

Reviewer #2: Yes

4. Is the manuscript presented in an intelligible fashion and written in standard English?

PLOS ONE does not copyedit accepted manuscripts, so the language in submitted articles must be clear, correct, and unambiguous. Any typographical or grammatical errors should be corrected at revision, so please note any specific errors here.

Reviewer #1: No

Reviewer #2: Yes

5. Review Comments to the Author

Please use the space provided to explain your answers to the questions above. You
may also include additional comments for the author, including concerns about dual
publication, research ethics, or publication ethics. (Please upload your review as
an
attachment if it exceeds 20,000 characters)

Reviewer #1: The work entitled “Perceived discrimination and contextual
problems among children and adolescents in Northern Chile" is of great
interest. The research is very stimulating; it contains new scientific knowledge and
provides comprehensive information for further development of this productive line
of research. However, I have some comments to make that should be addressed before I
recommend this manuscript for publication.

- In the introduction, authors should consider devoting some lines about psychological problems in children and adolescence. With this regard recent relevant research analyzing prevalence of emotional and behavioural problems at those ages should be introduced (Dalsgaard et al., 2020; Ortuño-Sierra, Aritio-Solana,
& Fonseca-Pedrero, 2018).

The inclusion of mental health studies diverts the focus from discrimination in relation to contextual problems. We have reviewed the articles, but they do not mention the contextual problems as to connect them with our article

- Authors should consider the following affirmation in the introduction: “Based on the aforementioned facts,
we believe this study could contribute significantly to reduce the effects of
discrimination in schools within the northernmost regions of Chile and other
applicable contexts”. First, this sentence would better fit in the discussion
section. Second I am not completely sure about the adequacy of such affirmation
considering the nature of the study. Authors may contribute relevant information to
comprehend the nature of the problema and from that prevent discrimination but they
dont know if that reduce significantly the effects of discrimination.

We rewording it to keep in the same section as part of relevance to investigate this topic

- In addition, the following sentence should be placed in the discussion section.

“The aim of the study was achieved, as perceived discrimination and ethnic identity were regarded”. With this regard, the objectives section should provide information about hypothesis.

We removed that sentence.

- In the participants section, authors may consider including the age distribution.

We added it

- In the instruments section, authors please check for grammatical mistakes in the following sentence: “It is a Likert-type scale consisted…”

We removed it

- In addition, authors should described the estimator when talking about evidences of internal consistency of the scores. Also, evidences of the MEIM-R are not provided.

We described MLR estimator in SEM, and added omega indices in each instrument

- Moreover, authors should consider explaining whether the instruments are self-reported or information could or have to be provided by parents, teacher and/or clinicals. This is relevant beacuse in the procedure it is not clear (please also consider modifying) if the students themselves completed the questionnaires or if the teachers or someone else provided information about the students.

We added it. Also, we expand procedure section

- With this regard, and assuming the students completed the questionnaires, have the authors consider that there are concerns raised about children under 11-10 years old answering self-reported questionnaires due to the lack of instrospection?

We explained that there are two SENA instruments. One for each group, specially address to be easy to understand and answer.

- Also, it seems after eading the procedure that the written consent was asked to the participants. I do not know how appropriate it is that under 18 and in this case children of 8,9,10 years old provide this kind of consent. In my opinion, parents, teachers or legal tutors should have asked for this aspect.

We added information in procedure section. First, we ask to parents. Then we ask student themselves. We think that is more respectful with the child’s and adolescents.

- I would also consider making the objectives more specifics and related to the results and conclusions. It is not till the results and the discussion section when one start realizing why the authors includen the SEM analysis.

We added three hypotheses to connect objective with analysis and results

Please also explain in the data analysis section the reference values for the goodnes-of-fit índices values instead of doing it in the results section.

We moved it

- Finally, authors should consider revising the writing when talking about internal consistency or validity in some parts of the paper. First of all, validity is not a property of the test but inferences of the scores, and also, there are sources or validity evidences, as it is reflected in the APA standards. Attending to this approach it would be more appropriate to talk about evidences of internal structure or evidences of relation with other variables or external variables. In addition, the reliability is not a characteristic of the test. It is more correct to talk about reliability of the scores or estimation of the reliability of the scores (Prieto & Delgado, 2010).

We rewording it

Reviewer #2: Lines 176, 183, 200, 220: please indicate the metric properties of the instruments and clarify if they have validation in your country. Not only Spanish translation.

We added it

Lines 262 to 263: It is not clear why you say that there is no statistically significant correlation between ethnic identity and problems with peers. In the correlation chart the information is different.

It was non-significant correlation. We remarked this again.
r= ,020

6. PLOS authors have the option to publish the peer review history of their article (what does this mean?). If published, this will include your full peer review and any attached files.

If you choose “no”, your identity will remain anonymous but your review may still be made public.

Do you want your identity to be public for this peer review? For information about this choice, including consent withdrawal, please see our Privacy Policy.

Reviewer #1: No

Reviewer #2: No

[NOTE: If reviewer comments were submitted as an attachment file, they will be attached to this email and accessible via the submission site. Please log into your account, locate the manuscript record, and check for the action link "View Attachments". If this link does not appear, there are no attachment files to be viewed.]

While revising your submission, please upload your figure files to the Preflight Analysis and Conversion Engine (PACE) digital diagnostic tool, https://pacev2.apexcovantage.com/. PACE helps ensure that figures meet PLOS requirements. To use PACE, you must first register as a user. Registration is free. Then, login and navigate to the UPLOAD tab, where you will find detailed instructions on how to use the tool. If you encounter any issues or have any questions when using PACE, please email us at figures@plos.org. Please note that Supporting Information files do not need this step.

In compliance with data protection regulations, you may request that we remove your personal registration details at any time. (Remove my information/details). Please contact the publication office if you have any questions.
