# Supplementary material for: Perceived discrimination and contextual problems among children and adolescents in northern Chile
Source: PLoS One. 2021 Feb 19;16(2):e0246998. doi: 10.1371/journal.pone.0246998 (PMC7894936; doi:10.1371/journal.pone.0246998)
Supplement: Attachment — Submitted filename: Response to Reviewers.pdf [file pone.0246998.s008.pdf]

Dear Editor and Reviewers:

Thank you for all the helpful comments. We present below our answers to each observation.

First, to test your hypotheses, it will be important for you to formulate your SEM more clearly in line with them. You are testing the relation between perceived discrimination and various problems that children have, and this requires that discrimination is the exogenous variable in your model and the two other variables (collective esteem and ethnic identification) are mediators between discrimination and your outcomes. Your models should reflect that. As they are currently presented, you have ethnic identification as exogenous and collective esteem as a mediator. Please redo you models so that both of the other two variables are mediators between discrimination and problems.

Re: we added two theoretical models in the beginning. The reason these models changes it was along with the data analysis, we noted that there is not a correlation between discrimination and ethnic identity. Then not makes sense to keep the original model. Anyway, if we run the original model, the result is the same because there is no significant relationship between discrimination and ethnic identity, and the arrows in the model only reflect significant relationships. Now we explain the process more clear.

Second, please add age and gender to the correlation matrices for both age groups so that one can see how these other exogenous variables are related to your survey measures.

RE: We can not add sex (binary) in a Pearson correlation matrix. Besides, we already test gender in MANOVA. We prefer to keep gender and age in MANOVA to show any interaction effect. Anyway, there is also a way to show if these variables need to be added to the SEM models.

Third, if your correlation matrices are correct, there is a dramatic difference between the younger and the older age groups in the way that discrimination is related to problems. It is negatively related in the younger group but positively in the older youth. This should be a major focus of your analyses and interpretations. I do not see any indication that you have recognized this in any of your models.

Re: We apologize for that. It was a mistake in primary values. We already correct sings.

Fourth, you have a major problem in regard to clustering by school. Your analyses collapse over schools and therefore it is not clear how much is due to school differences versus individual differences. You can remove school differences before analyzing the data so that you are more certain that the relations you are seeing are due to individual differences and not differences between schools. Or you can include dummy variables for schools in your models so that they are controlled. But you have to do something to handle this problem.

RE: Thanks for this suggestion. Like three types of school are clearly not equivalent groups (private school are 5% approximate in bot samples, don't makes sense to compare in this way).

We have the option to consider in the binary way public (only public) and No public school (subsidized and particular). But these no necessary reflect vulnerability since several subsidized schools have not cost for children. Then we added now a vulnerability school variable in both models, on basis a national vulnerability index. We added index vulnerability information in the instruments section. It is better tan classification by public or not public school. We estimate both (school type and school vulnerability), but the vulnerability criterion was clearly more significant. The models keep similar values and good fit.

I do not understand what you mean by "unitary model of contextual problems" in line 385. Are you collapsing over all of the problem scales or just using the context outcome?

RE: There are three specific contextual problems scales in SENA: family, schools, and peers. Also, all these are combined in one global index of contextual problems. Unitary models are the last case, but now we changed the name to global model along with all manuscript. Specific models are the first case. Now we added two figures of theoretical models to improve clarity. Along with the paper, we try to decide which one is a better model to consider.

I think it would be clearer if you called your ethnic identity variable "ethnic identification," which makes it clearer that it is not actual identity that you are measuring but how strongly youth identify with their identity.

Re: we try to keep the original sense of the scale, but we can add this topic in the discussion

It is not clear how the multiple imputation was involved in the analyses. How did you use that?

Re: Mulitple imputations and FIML are asymptotically equivalent. We chose to use MI because is robust in the case to non-normal distribution according to Nakagawa (2015) and Enders(2010). all variables were used in each sample to perform multiple imputation of missing values

Was it for the MANOVA? It is not clear how it would be used in the SEM.

RE: In the previous review, MANOVA was requested to establish whether age and gender are within or outside the models in the primary and secondary groups. SPSS also allows multiple imputation, and we perform it using the same criteria as in MPLUS, with all variables and with 7 datasets.

It is also not clear how you used the effect size measure in lines 315-317.

RE: we remove the effect size for MANOVA since it is not relevant in this case because just a significant effect is enough to consider one variable in the subsequent SEM model.

Nor is it clear how you used the missing at random test in lines 318-322.

RE: Little's MCAR test was used to examined missing values. It was significant in both samples. Anyway, Enders(2010) consider that is not enough exact test.

A clearer explanation of how much data were missing would be helpful. With SEM, maximum likelihood imputation is usually used to handle missing data.

Re: we added this: the maximum number of unanswered items for each participant was less than 20% in both samples. 28 cases in primary and 36 cases in secondary had all the items of perceived discrimination as missing values.

Multiple imputations and full information maximum likelihood (FIML) are asymptotically equivalent. We chose to use MI because it is better with non-normal distribution, according to Nacagawa (2015) and Enders(2010). All variables were used in each sample to perform multiple imputation of missing values

Please describe the difference between Public and Subsidized schools.

RE: Public schools are fully funded by the government. While government-subsidized schools only receive partial funding to operate, they are free to charge school fees per pupil. Private schools do not receive any funding; they charge school fees per pupil.

Please provide a reference for the omega statistic.

RE: We added Hayes & Coutts (2020) reference in the data analysis section

Only scales that use the agree-disagree format are truly Likert scales. Other types of responses are merely rating scales (e.g., never to every day).

RE: we modified all.

It is not clear why you say that the self-esteem scale is a self-report instrument (line 217) when all of your assessments involve self-report.

RE: All instrument we used were self-report, but we prefer to specify in each one to be clear

It is not clear what you mean in lines 228-229 about "adjusted in relation to nationality."

RE: collective self-esteem was considerer in national level. We added one example of ítem. The initial instruction of this instruments was "Depending on which nationality you consider yourself, mark your grade according to the following statements."

You should also remove all statements of causal direction in your description of the findings. Rather than saying a variable had a direct effect, you can say that it was directly related. You are also using regression in your models, so path weights are regression weights and not correlations.

RE: we modified all.

Also, please describe how you tested indirect relations. This is usually done with bootstrapping.

RE: We use the model indirect effect syntaxis in MPLUS. Boostraping is not available for multiple imputation.

Finally, you still have issues with appropriate use of English. I highlighted some places where the English is not clear.

RE: we review and rewrite all these
