# Supplementary material for: Perceived discrimination and contextual problems among children and adolescents in northern Chile
Source: PLoS One. 2021 Feb 19;16(2):e0246998. doi: 10.1371/journal.pone.0246998 (PMC7894936; doi:10.1371/journal.pone.0246998)
Supplement: Attachment — Submitted filename: Response to Reviewers.docx [file pone.0246998.s006.docx]

```
PONE-D-20-05463
Perceived discrimination and contextual problems among children and adolescents in
Northern Chile
PLOS ONE

Dear Jerome An&iacute;bal,

Thank you for submitting your manuscript to PLOS ONE. After careful consideration,
we feel that it has merit but does not fully meet PLOS ONE&rsquo;s publication
criteria as it currently stands. Therefore, we invite you to submit a revised
version of the manuscript that addresses the points raised during the review
process.


We would appreciate receiving your revised manuscript by 16/07/2020. When you are
ready to submit your revision, log on to https://www.editorialmanager.com/pone/ and
select the &#39;Submissions Needing Revision&#39; folder to locate your manuscript
file.

If you would like to make changes to your financial disclosure, please include your
updated statement in your cover letter.

To enhance the reproducibility of your results,
we recommend that if applicable you deposit your laboratory protocols in
protocols.io, where a protocol can be assigned its own identifier (DOI) such that it
can be cited independently in the future. For instructions see:
http://journals.plos.org/plosone/s/submission-guidelines#loc-laboratory-protocols

Please include the following items when submitting your revised manuscript:A
rebuttal letter that responds to each point raised by the academic editor and
reviewer(s). This letter should be uploaded as separate file and labeled
&#39;Response to Reviewers&#39;.A marked-up copy of your manuscript that highlights
changes made to the original version. This file should be uploaded as separate file
and labeled &#39;Revised Manuscript with Track Changes&#39;.An unmarked version of
your revised paper without tracked changes. This file should be
uploaded as separate file and labeled &#39;Manuscript&#39;.
Please note while forming your response, if your article is accepted, you may have
the opportunity to make the peer review history publicly available. The record will
include editor decision letters (with reviews) and your responses to reviewer
comments. If eligible, we will contact you to opt in or out.
We look forward to receiving your revised manuscript.

Kind regards,

Eduardo Fonseca-Pedrero, PhD
Academic Editor
PLOS ONE

Journal Requirements:When submitting your revision, we need you to address these
additional requirements.
1. Please ensure that your manuscript meets PLOS ONE&#39;s style requirements,
including those for file naming. The PLOS ONE style templates can be found at
https://journals.plos.org/plosone/s/file?id=wjVg/PLOSOne_formatting_sample_main_body.pdf
and
https://journals.plos.org/plosone/s/file?id=ba62/PLOSOne_formatting_sample_title_authors_affiliations.pdf
2. We note that you have stated that you will provide repository information for
your data at acceptance. Should your manuscript be accepted for publication, we will
hold it until you provide the relevant accession numbers or DOIs necessary to access
your data. If you wish to make changes to your Data Availability statement, please
describe these changes in your cover letter and we will update your Data
Availability statement to reflect the information you provide.
3. Please use periods &quot;.&quot; instead of commas&quot;,&quot; for decimal points.
4. Please include additional information regarding the survey or questionnaire used
in the study and ensure that you have provided sufficient details that others could
replicate the analyses. For instance, if you developed a questionnaire as part of
this study and it is not under a copyright more
restrictive than CC-BY, please include a copy, in both the original language and
English, as Supporting Information. Please clarify whether you used a Spanish
version of the MEIM-R and if so how it was validated.

Additional Editor Comments (if provided):

The work entitled &quot;Perceived discrimination and contextual problems among
children and adolescents in Northern Chile&rdquo; is a good research paper. However,
I have a few comments to make that should be addressed before I recommend this
manuscript for publication to PLOS ONE:
Dear Editor and reviewers:

We are grateful of all comments. We decide to divide the sample in two groups, primary and secondary, since we used different SENA age version in each one. Even if both SENA version share more than 70% items, slight differences in the rest can affect the analysis, and the secondary version has one item more.

1.- Please, add empirical information in the abstract.

It was adding now

2.- Please, add an operative definition of the main constructs used.

There is not a cut-off point for to establish enough collective self-esteem or ethnic identity. Then it was not specified. SENA has cut-off for four risk zones, but it was not used because a continuous variable can show clearer the relationship.

3.- In English, a decimals point is used, not comma.

It was changed

4.- Please, add the main hypothesis at the end of the introduction.

We added three hypotheses

5.- Add more information about sample and sampling procedure as well as others variables like: % by age, socio-economic level, IQ, previous mental disorders, etc. Add all the sample information in the method
section, not as a results.

It was added all information and change the position in the document. We don’t ask socioeconomic level to child’s because the primary group probably don’t know

6.- Do you have any information about non-response? Describe inclusion/exclusion criteria if part of the data was excluded from the analysis.

We added criteria

7.- Were outliers removed from the data? Which method did you use to deal with
missing data in the analyses? What variables are related to missing data?

We identified out-layers in both groups, but we dint remove. We explain the differences between include or not at the end of result section.
The effects of removing the out-layers are considered. The vast majority of the relationships are maintained and vary in a range of 3-5%. Only one relationship ceases to be significant, ethnic identity on school problems in primary group. In sum, this means that relationships are not generated by the presence of out-layers. In any case, by removing them and recalculating the Z-scores, new out-layers appear. Since the MLR estimator that is resistant to non-normality has been chosen, it is decided to keep all the cases in the sample.

8.- Add information about the psychometric properties of the measures used in this study. In addition, add Omega values, not Cronbach. Cronbach alpha has a lot of limitations from a psychometric point of view.

We added all information about each scale and omega indices for all

9.- Add subheadings in the results section (as function of the goals).
We added subheadings in this section

10.- Add more information about mediation analyses performed.

We added all information

11.- Please, before to SEM analysis, compute a MANOVA or MANCOVA in mean comparisons by gender and age. Add effect sizes in mean comparisons.

We added a MANOVA and effect sizes

12.- Please, add other godness of fit indices, eg.: TLI, BIC, IC RMSEA 90%, etc. Add information about
goodness of fit indices. Test model assumptions (e.g., normality).

We added information about normality, and more fit indices

13.- Add more limitations (e.g., self-reports, no scale to test social desirability).

We added

14.- Check English grammar.

We reviewed it

[Note: HTML markup is below. Please do not edit.]

Reviewers&#39; comments:

Reviewer&#39;s Responses to Questions

Comments to the Author

1. Is the manuscript technically sound, and do the data support the conclusions?

The manuscript must describe a technically sound piece of scientific research with
data that supports the conclusions. Experiments must have been conducted rigorously,
with appropriate controls, replication, and sample sizes. The conclusions must be
drawn appropriately based on the data presented.

Reviewer #1: Partly

Reviewer #2: Yes

2. Has the statistical analysis been performed appropriately and rigorously?

Reviewer #1: Yes

Reviewer #2: Yes


3. Have the authors made all data underlying the findings in their manuscript fully
available?

The PLOS Data policy requires authors to make all data underlying the findings
described in their manuscript fully available without restriction, with rare
exception (please refer to the Data Availability Statement in the manuscript PDF
file). The data should be provided as part of the manuscript or its supporting
information, or deposited to a public repository. For example, in addition to
summary statistics, the data points behind means, medians and variance measures
should be available. If there are restrictions on publicly sharing data&mdash;e.g.
participant privacy or use of data from
a third party&mdash;those must be specified.

Reviewer #1: Yes

Reviewer #2: Yes


4. Is the manuscript presented in an intelligible fashion and written in standard
English?

PLOS ONE does not copyedit accepted manuscripts, so the language in submitted
articles must be clear, correct, and unambiguous. Any typographical or grammatical
errors should be corrected at revision, so please note any specific errors here.

Reviewer #1: No

Reviewer #2: Yes


5. Review Comments to the Author

Please use the space provided to explain your answers to the questions above. You
may also include additional comments for the author, including concerns about dual
publication, research ethics, or publication ethics. (Please upload your review as
an
attachment if it exceeds 20,000 characters)

Reviewer #1: The work entitled &ldquo;Perceived discrimination and contextual
problems among children and adolescents in Northern Chile&quot; is of great
interest. The research is very stimulating; it contains new scientific knowledge and
provides comprehensive information for further development of this productive line
of research. However, I have some comments to make that should be addressed before I
recommend this manuscript for publication.

- In the introduction, authors should consider devoting some lines about
psychological problems in children and adolescence. With this regard recent relevant research analyzing prevalence of emotional and behavioural problems at those ages should be introduced (Dalsgaard et al., 2020; Ortu&ntilde;o-Sierra, Aritio-Solana,
&amp; Fonseca-Pedrero, 2018).

The inclusion of mental health studies diverts the focus from discrimination in relation to contextual problems. We have reviewed the articles, but they do not mention the contextual problems as to connect them with our article

- Authors should consider the following affirmation in the introduction:
&ldquo;Based on the aforementioned facts,
we believe this study could contribute significantly to reduce the effects of
discrimination in schools within the northernmost regions of Chile and other
applicable contexts&rdquo;. First, this sentence would better fit in the discussion
section. Second I am not completely sure about the adequacy of such affirmation
considering the nature of the study. Authors may contribute relevant information to
comprehend the nature of the problema and from that prevent discrimination but they
dont know if that reduce significantly the effects of discrimination.

We rewording it to keep in the same section as part of relevance to investigate this topic

- In addition, the following sentence should be placed in the discussion section.
&ldquo;The aim of the study was achieved, as perceived discrimination and ethnic identity were regarded&rdquo;. With this regard, the objectives section should provide information about hypothesis.

We removed that sentence.

- In the participants section, authors may consider including the age distribution.

We added it

- In the instruments section, authors please check
for grammatical mistakes in the following sentence: &ldquo;It is a Likert-type scale
consisted&hellip;&rdquo;

We removed it

- In addition, authors should described the estimator when talking about evidences of internal consistency of the scores. Also, evidences of the MEIM-R are not provided.

We described MLR estimator in SEM, and added omega indices in each instrument

- Moreover, authors should consider explaining whether the instruments are
self-reported or information could or have to be provided by parents, teacher and/or
clinicals. This is relevant beacuse in the procedure it is not clear (please also
consider modifying) if the students themselves completed the questionnaires or if
the teachers or someone else provided information about the students.

We added it. Also, we expand procedure section

- With this regard, and assuming the students completed the questionnaires, have the authors consider that there are concerns raised about children under 11-10 years old
answering self-reported questionnaires due to the lack of instrospection?

We explained that there are two SENA instruments. One for each group, specially address to be easy to understand and answer.

- Also, it seems after eading the procedure that
the written consent was asked to the participants. I do not know how appropriate it
is that under 18 and in this case children of 8,9,10 years old provide this kind of
consent. In my opinion, parents, teachers or legal tutors should have asked for this
aspect.

We added information in procedure section. First, we ask to parents. Then we ask student themselves. We think that is more respectful with the child’s and adolescents.

- I would also consider making the objectives more specifics and related to the
results and conclusions. It is not till the results and the discussion section when
one start realizing why the authors includen the SEM analysis.

We added three hypotheses to connect objective with analysis and results

Please also explain
in the data analysis section the reference values for the goodnes-of-fit
&iacute;ndices values instead of doing it in the results section.

We moved it

- Finally, authors should consider revising the writing when talking about internal
consistency or validity in some parts of the paper. First of all, validity is not a
property of the test but inferences of the scores, and also, there are sources or
validity evidences, as it is reflected in the APA standards. Attending to this
approach it would be more appropriate to talk about evidences of internal structure
or evidences of relation with other variables or external variables. In addition,
the reliability is not a characteristic of the test. It is more correct to talk
about reliability of the scores or estimation of the reliability of the scores
(Prieto &amp; Delgado, 2010).

We rewording it


Reviewer #2: Lines 176, 183, 200, 220: please indicate the metric properties of the
instruments and clarify if they have validation in your country. Not only Spanish
translation.

We added it

Lines 262 to 263: It is not clear why you say that there is no statistically
significant correlation between ethnic identity and problems with peers. In the
correlation chart the information is different.

 It was non-significant correlation. We remarked this again.
r= ,020


6. PLOS authors have the option to publish the peer review history of their article
(what does this mean?). If published, this will include your full peer review and
any attached files.


If you choose &ldquo;no&rdquo;, your identity will remain anonymous but your review
may still be made public.


Do you want your identity to be public for this peer review? For information about
this choice, including consent withdrawal, please see our Privacy Policy.

Reviewer #1: No

Reviewer #2: No


[NOTE: If reviewer comments were submitted as an attachment file, they will be
attached to this email and accessible via the submission site. Please log into your
account, locate the manuscript record, and check for the action link &quot;View
Attachments&quot;. If this link does not appear, there are no
attachment files to be viewed.]


While revising your submission, please upload your figure files to the Preflight
Analysis and Conversion Engine (PACE) digital diagnostic tool,
https://pacev2.apexcovantage.com/. PACE helps ensure that figures meet PLOS
requirements. To use PACE, you must first register as a user. Registration is free.
Then, login and navigate to the UPLOAD tab, where you will find detailed
instructions on how to use the tool. If you encounter any issues or have any
questions when using PACE, please email us at figures@plos.org. Please note that
Supporting Information files do not need this step.


 In compliance with data protection regulations, you may request that we remove your
personal registration
details at any time.  (Remove my information/details). Please contact the
publication office if you have any questions.
```
