# Supplementary material for: Perceived discrimination and contextual problems among children and adolescents in northern Chile
Source: PLoS One. 2021 Feb 19;16(2):e0246998. doi: 10.1371/journal.pone.0246998 (PMC7894936; doi:10.1371/journal.pone.0246998)
Supplement: Attachment — Submitted filename: Response to reviewers.docx [file pone.0246998.s010.docx]

Dear editor and reviewers:

Thanks for all your comments. We present a new version of manuscript.

Although you have made improvements in the readability of the paper and have clarified some of the concerns that were raised in the last review, the paper still has serious limitations. I think the results do show that discrimination directed toward students is related to various problems in your schools, but it is not clear that this is a problem with individuals as opposed to differences between schools. All of your findings could result from what are known as between-group differences, which would have very different implications from differences due to individual experiences across

schools. Unless you can clarify this in your next revision, should you choose to submit one, it will not be possible to publish this in PLOS ONE. You may want to consult with a data analyst who is familiar with this distinction and who can conduct the necessary analyses.

RE: We performed a multilevel analysis of two levels. it was necessary to do a path analysis instead of a SEM. The reason was that it is not recommended that there are more parameters than clusters. The results are very similar to those found with SEM.

In addition, you still have a contradiction in your correlation matrices between primary and secondary students in regard to the relation between collective esteem and discrimination. You said this was a mistake, but it&#39;s still there in the table.

Re: We remade the entire table, adding age and sex as requested.

I still don&#39;t understand why age and gender are not in the orrelation matrices but are in the SEM. There is no reason not to have them in the correlations.

Re: We add both.

I must ask you again to not label ethnic identification as ethnic identify. This will confuse most readers, as ethnic identity is merely how one labels on ethnicity.

Your measure is more of an esteem measure, which is why it is related to your measure of collective esteem and why it behaves similarly.

Re: we change ethnic identity by ethnic identification

I again must ask you to refrain from using causal language in describing your results. Also, I asked you to label the relations in the model as regression coefficients rather than correlations. But you still refer to them as r values.

Re: we change the causal language of the results and conclusions. Also we use the language according to regression coefficients

Finally, I see no reason to present two sets of analyses for the collective problems and the individual components of that measure. It is sufficient to present the results broken out by the subscales, especially since they are so highly related to the overall measure and it is the difference in these that distinguishes your two age groups.

Re: We only present specific models of contextual problems. We eliminate models of global contextual problems.

In view of these continued concerns, we invite you to submit a revised version of the manuscript that addresses the points raised above. I again attach a highlighted version of your manuscript where your language needs attention.

Re: We made changes to the language in the sections indicated

We Will looking fodward your answer.

Best regards.

Phd. Jerome Flores Jara

Centro de Justicia Educacional

Universidad de Tarapacá
