# Supplementary material for: Perceived discrimination and contextual problems among children and adolescents in northern Chile
Source: PLoS One. 2021 Feb 19;16(2):e0246998. doi: 10.1371/journal.pone.0246998 (PMC7894936; doi:10.1371/journal.pone.0246998)
Supplement: Attachment — Submitted filename: Response to Reviewers.docx [file pone.0246998.s011.docx]

Dear Editor and Reviewers:

Thank you for your valuable comments. We have reviewed the manuscript and made further modifications that we hope will be sufficient.

We have changed the initial theoretical model, since by mistake we had the original general model. Now the specific theoretical model remains.

We have changed the number in table 3. It was an error to post under number 2 on page 18.

We check with a native American speaker all modifications in the paper.

Thank you for responding to my concerns in your last submission. I only have a few remaining concerns regarding the way you describe some of your methods, procedures, and findings.

In the Abstract, I’ve highlighted some places where things could be clearer (see attached). “The sample was divided into primary and secondary education groups” would be clearer. “For data analysis, we tested a path analytic model at both the within and between levels to account for the relations between variables.”

Re: we changed each one

Not sure what you mean by “and all were optimally adjusted.”

Re: we changed for optimum goodness fit

Line 291: you continue to use “ethnic identity”

Re: we changed We had kept it because this is the original explanation of the scale

Line 299: please fix the heading

Re: we changed

Line 301: please fix the wording

Re: we changed

Lines 352-353: this is confusing. why report the missing at random test if you are going to ignore it?

Re: we eliminated it to avoid confusion. It was reported as a requirement of the first revisions

Line 356: not sure what you are saying about "concentrate missing values"

Re: it was eliminated, now we only refer to the missing values over the total score

Lines 358: your description of missing data is confusing. How much missing data did you have? Was it only 28 and 36 cases or was it 45.3% and 36.2%? If the latter, that’s a lot. And if so, I would report what happens if you drop the missing cases and only analyze using list-wise deletion.

Re: we added a paragraph explaining what happens if we only consider the full cases.

Line 359: do you mean the scale was the last one in the survey?

Re: Yes, now we changed

Table 2: please label the collective self-esteem variable the same way in both the figures and the table.

Re: we changed all tables to match with figures

Line 445: is that p value correct?

Re: we changed. It was a mistake

Line 544: “visibly” does not make sense.

Re: we changed the wording

Line 563: please fix the wording

Re: we changed the wording

Line 656: not clear what you are referring to by “second level”. Please refer to this as the between group level, if that’s what you mean.

Re: we changed the wording

Again thank you for your comments.

We look forward to hearing from you

Dr. Jerome Flores Jara

Centro de Justicia Educacional

Universidad de Tarapacá
